# Chronic cerebral hypoperfusion exacerbates amyloid and tau pathology by impairing glymphatic transport via AQP4‐ and VEGF‐mediated pathways: insights from a vascular to mixed‐type dementia model

**DOI:** 10.1002/alz.71290

**Published:** 2026-03-17

**Authors:** Jia‐Hung Chen, Ching‐Wen Chang, You‐Yin Chen, Chih‐Hao Yang, Wen‐Bin Yang, Yi‐Chen Hsieh, Yao‐Wen Liang, Ssu‐Ju Li, Chaur‐Jong Hu

**Affiliations:** ^1^ Department of Neurology Taipei Medical University‐Shuang Ho Hospital New Taipei Taiwan; ^2^ Department of Neurology School of Medicine College of Medicine Taipei Medical University Taipei Taiwan; ^3^ Ph.D. Program in Medical Neuroscience College of Medical Science and Technology Taipei Medical University Taipei Taiwan; ^4^ Department of Biomedical Engineering National Yang Ming Chiao Tung University Taipei Taiwan; ^5^ Department of Pharmacology School of Medicine College of Medicine Taipei Medical University Taipei Taiwan; ^6^ Research Center for Neuroscience Taipei Medical University Taipei Taiwan

**Keywords:** aquaporin‐4, chronic cerebral hypoperfusion, dementia, glymphatic, phosphorylated tau 217, vascular endothelial growth factor

## Abstract

**INTRODUCTION:**

Chronic cerebral hypoperfusion (CCH) is a major contributor to cognitive impairment; however, its underlying mechanisms remain poorly understood.

**METHODS:**

We investigated CCH‐induced glymphatic dysfunction and neurodegeneration in amyloid precursor protein (APP)/presenilin 1 (PS1) and wild‐type mice. Glymphatic transport was assessed using contrast‐enhanced magnetic resonance imaging (MRI) and real‐time femoral vein imaging. Aquaporin‐4 (AQP4) polarization and amyloid beta (Aβ)/phosphorylated tau 217 (p‐tau217) accumulation were examined by immunofluorescence staining. Single‐cell RNA sequencing (scRNA‐seq) identified molecular mechanisms and pathways.

**RESULTS:**

CCH impaired glymphatic clearance by reducing AQP4 polarization, resulting in Aβ and p‐tau217 accumulation. scRNA‐seq revealed downregulation of vascular endothelial growth factor (VEGF), Rho GTPase, and integrin–actin signaling pathways. Restoring vascular tone with adrenergic receptor blocker normalized VEGF localization and vascular pulsatility/resistance, improved glymphatic clearance, and rescued cognitive function.

**DISCUSSION:**

CCH impairs glymphatic function through AQP4 depolarization and VEGF suppression, causing toxic protein accumulation. Restoring vascular tone rescued cognition, establishing a mechanistic link between vascular dysfunction and neurodegeneration in cognitive impairment.

## Highlights

- We investigated the role of CCH in regulating the glymphatic system and its contribution to cognitive decline.
- Glymphatic clearance was markedly impaired following BCCAO, as evidenced by reduced wash‐in and washout kinetics.
- AQP4 depolarization after BCCAO led to Aβ and phosphorylated tau accumulation.
- Downregulation of VEGF signaling, Rho GTPase, and integrin–actin cytoskeletal pathways was observed following BCCAO.

## 1 BACKGROUND

Cerebral blood flow (CBF) is essential for neuronal activity and brain homeostasis.^1^, ^2^ Chronic cerebral hypoperfusion (CCH), characterized by reduced blood flow to the brain, is recognized as a major contributor to neurodegeneration.^3^, ^4^ CCH has been implicated in the pathogenesis of various forms of dementia, including Alzheimer's disease (AD) and vascular dementia.^5^, ^6^ Mixed‐type dementia, which typically involves both AD and vascular pathology, is a common form of dementia that is also linked to CCH.^7^ Sustained reductions in CBF can result in amyloid beta (Aβ) and tau accumulation, neuroinflammation, and blood–brain barrier dysfunction, leading to cognitive decline.^8^, ^9^, ^10^ However, despite the fact that CCH causes neuronal damage, synaptic loss, and cognitive impairment, the mechanisms by which CCH drives pathogenic processes in AD, vascular dementia, and mixed‐type dementia are poorly understood.

CCH may disrupt glymphatic function, a major brain clearance pathway that facilitates the removal of interstitial metabolic waste.^11^, ^12^ Efficient glymphatic function is crucial for brain homeostasis and effective protein clearance. Some proteins are pathological, playing key roles in neurodegeneration. Therefore, effective glymphatic clearance is important for preventing the accumulation of pathological proteins.^13^ Previous studies demonstrated that dysfunction of the glymphatic system is associated with the accumulation of Aβ, a hallmark of AD pathology.^14^, ^15^ Enlarged perivascular space, which is indicative of glymphatic system dysfunction, has also been observed in patients with cerebral small vessel disease, which is the most common cause of vascular dementia.^16^, ^17^ Although the glymphatic system is increasingly recognized as a therapeutic target in dementia,^18^ it remains unclear whether and by what mechanisms CCH impairs its function.

To address this knowledge gap, we investigated the effects of CCH on glymphatic transport efficiency and cognitive outcomes using mouse models of AD, vascular dementia, and mixed‐type dementia. Specifically, we explored the effects of CCH on glymphatic function and neurodegeneration, particularly in the context of proteinopathy. In this study, we utilized contrast‐enhanced magnetic resonance imaging (MRI) to evaluate glymphatic clearance and further investigated the association between aquaporin‐4 (AQP4) depolarization, CBF hemodynamic changes, and glymphatic function. Additionally, we employed single‐cell RNA sequencing (scRNA‐seq) to explore cell‐type‐specific transcriptional changes related to glymphatic dysfunction and the effects of CCH. Our findings clarify how CCH‐induced vascular changes contribute to glymphatic system dysfunction, proteinopathy, and cognitive decline.

## 2 METHODS

### 2.1 Animal model

Five‐month‐old APPswe/PSEN1dE9 transgenic mice (AD mice) were purchased from Jackson Laboratory (Bar Harbor, ME, USA). Wild‐type (WT) littermates were used as controls. The mice were housed in controlled environments (temperature: 22°C ± 1°C; humidity: 55% ± 10%; photoperiod: 12‐h light/dark, lights on at 7:00 a.m.), with ad libitum access to food and water. All animal care and experimental protocols adhered to relevant guidelines for the care and use of laboratory animals. The study protocol was approved by the Ethics Committee of Taipei Medical University. The animal use protocol was approved by the Institutional Animal Care and Use Committee (approval no. LAC‐2021‐0123; approval date: January 4, 2017).

### 2.2 Bilateral common carotid artery occlusion procedure

We employed a modified bilateral common carotid artery occlusion (BCCAO) model consisting of permanent ligation of the right common carotid artery (CCA) combined with transient ligation of the left CCA. Mice were randomly assigned to receive either BCCAO or sham surgery, resulting in four experimental groups: WT mice with BCCAO (WT‐BCCAO), WT mice with sham surgery (WT‐sham), AD mice with BCCAO (AD‐BCCAO), and AD mice with sham surgery (AD‐sham). BCCAO was performed in two steps, as described in our previous study.^19^ The first step involved permanently ligating the right CCA. The second step, which was performed 1 week later, involved transiently ligating the left CCA for 30 min. After the procedure, the mice were placed on a heating pad until they regained consciousness (approximately 30 min). Once awake, the mice were returned to their cages. Mice with CBF reductions of at least 30% after 3 months were included in the analysis.

**RESEARCH IN CONTEXT**

1. **Systematic review**: We conducted a literature review in PubMed using keywords related to CCH, glymphatic system, and cognitive impairment. Most studies indicated that cognitive decline was associated with CCH in both AD and vascular dementia, likely through impaired glymphatic function; however, the underlying mechanisms remain inadequately understood.
2. **Interpretation**: Our findings support the hypothesis that CCH impairs glymphatic function by reducing AQP4 polarization, leading to the accumulation of toxic proteins and subsequent cognitive impairment. This mechanism appears to be driven by the downregulation of VEGF signaling, which has not been thoroughly investigated.
3. **Future directions**: This study proposes a framework linking vascular insufficiency to glymphatic dysfunction. Further investigations aimed at preserving vascular health and restoring AQP4 polarization and VEGF signaling are required to prevent or mitigate cognitive decline.

### 2.3 Novel object recognition test

A novel object recognition test was performed in three phases: habituation, sample, and test phases. In the habituation phase, mice were allowed to explore an empty arena for 30 min over a period of 3 days (10 min per day). This allowed the mice to become familiar with the arena. Next (in the sample phase), two identical objects were placed symmetrically within the arena, and the mice were placed at opposite walls and allowed to freely explore the objects for 10 min. The time the mice spent interacting with each object was recorded. In the test phase, one of the objects was replaced with a novel object. After 3 h, the mice were reintroduced to the arena. The mice were allowed to explore the arena for 10 min. To ensure consistency, the arena and objects were cleaned with 70% ethanol between trials. Behavioral performance was automatically tracked using a video system. Object recognition index values were calculated by dividing the time that the mice spent exploring the novel object by the total time spent exploring both novel and familiar objects.

### 2.4 Morris water maze test

A Morris water maze test was conducted using a circular pool with a diameter of 100 cm and a depth of 50 cm, maintained at a temperature of 20°C to 22°C. The pool was divided into four quadrants, each marked with distinct visual cues. A hidden platform was positioned 1.5 cm below the surface of the water in the upper left quadrant. Each mouse received six training sessions per day over 3 days. The training involved each mouse being placed in the pool from a different starting position and being given 60 s to locate the hidden platform. Mice that failed to find the platform within 60 s were placed on the platform for 20 s. A probe trial was then performed. Each mouse was placed in the pool's lower right quadrant, and the time taken to reach the platform was recorded.

### 2.5 Measurement of CBF, pulsatility index, and resistance index

CBF was measured using a laser speckle imaging system (RFLSI ZW; RWD Life Science, China) at three time points: before BCCAO, 1 month after it, and 3 months after it. The mice were anesthetized with 2% isoflurane. The hair on the scalp of each mouse was shaved. A midline incision was made. CBF was measured through the skull. The imaging system was set to the high‐speed mode (120 fps). The system recorded CBF for 20 s. CBF values for each mouse were recorded and plotted as a waveform. The pulsatility index was calculated using the following equation: pulsatility index = [peak systolic flow − end diastolic flow]/mean flow. Resistance index was calculated using the following equation: resistance index = [peak systolic flow − end diastolic flow]/peak systolic flow.^20^

### 2.6 Glymphatic function evaluation

To facilitate contrast agent delivery into the cisterna magna, mice were positioned in the supine position within the scanner.^21^ A micro‐infusion pump (KDS 100; KD Scientific, Holliston, MA, USA) located outside the scanner was connected to a pre‐implanted PE10‐glass composite micropipette, which was used to administer the agent via a micro‐syringe through the micro‐infusion pump system. Gadobutrol (Gd‐DOTA, Gadovist™; 1.0 mmol/mL, Bayer AG, Leverkusen, Germany) was first diluted 1:20 with sterile saline for further infusion into the cisterna magna. All MRI experiments were conducted on a 7T preclinical scanner (PharmaScan 70/16 US; Bruker, Wissembourg, France) equipped with 760 mT/m magnetic field gradients (B‐GA9S HP). A 72‐mm‐volume resonator was used for radiofrequency transmission, and a 2.3‐cm‐inner‐diameter mouse brain surface coil was used for signal reception. Image acquisition and scanner control were performed using ParaVision 6.0 software (Bruker, Wissembourg, France).

MRI scanning followed a standardized workflow comprising sequential B_1_^+^ field mapping, T_1_ mapping, contrast infusion, and post‐contrast dynamic imaging. First, B_1_^+^ field inhomogeneity was assessed using a double‐angle Rapid Acquisition with Relaxation Enhancement (RARE) sequence (repetition time [TR] = 10,000 ms, echo time [TE] = 22 ms, RARE factor = 4) with flip angles of 70° and 140°, acquired over 36 slices (slice thickness = 0.3 mm, gap = 0.2 mm) at an in‐plane resolution of 0.24 mm/pixel. Pre‐contrast T_1_ mapping was then performed using a variable flip angle (VFA) spoiled gradient recalled echo (VFA‐SPGR) sequence (TR = 16 ms, TE = 3 ms, NEX = 1), with six flip angles (2°, 5°, 10°, 15°, 20°, 30°), a 100 × 100 × 100 matrix, and isotropic voxel size of 0.18 mm. Following baseline imaging, 20 uL Gd‐DOTA was infused into the cisterna magna at 1 uL/min over 30 min using a micro‐infusion pump. Post‐contrast T_1_ maps were subsequently acquired at 30, 60, 90, 120, 180, 240, and 300 min after infusion using the same VFA‐SPGR parameters. A reference phantom containing 0.1 mM Gd‐DOTA was included within the field of view to facilitate signal normalization across all time points.

After acquisition of T_1_‐weighted images and VFA scans, voxel‐wise T_1_ mapping was performed using the variable flip angle SPGR method with three‐parameter non‐linear fitting of the SPGR signal model, as established in quantitative MRI protocols.^22^, ^23^ Concentration maps of Gd‐DOTA were derived by applying known relaxivities in brain tissue^21^, ^24^ and validated in phantom studies.^25^ A 0.1 mM Gd‐DOTA reference phantom was used for normalization across time points. To reduce noise and mitigate motion‐induced artifacts, voxel‐level signal dynamics were clustered using a data‐driven hierarchical k‐means approach.^26^ The first derivative of each voxel's time‐signal curve (TSC) was used as the clustering feature to capture temporal similarity in tracer kinetics. Clusters were iteratively subdivided if their voxel count exceeded 100 and their within‐cluster inconsistency (WCI) exceeded 0.125, computed based on Euclidean distances between derivative signals. Each final cluster yielded a representative TSC, which was used for modeling.

Quantitative assessment of glymphatic transport employed a two‐compartment exchange model,^21^, ^26^ in which rate constants K_1_ (influx), K_2_ (efflux), and reversible exchange terms K_3_/K_4_ describe tracer kinetics between blood, tissue, and interstitial compartments. These parameters were fitted to representative TSCs generated from clustered voxel groups using local input functions using forward Euler integration and weighed non‐linear least squares.^26^ A hybrid optimization scheme combining GlobalSearch and fmincon (MATLAB R2023b, Optimization Toolbox™) ensured convergence to global minima under non‐negativity constraints for all rate constants. Model goodness of fit was evaluated by overlaying predicted curves on experimental TSCs and inspecting residual distributions.

Following earlier MRI‐based glymphatic kinetic modeling studies in rodents^26^, ^27^, ^28^, ^29^ and recent quantitative imaging advances,^30^ wash‐in and washout rates and retention ratios for the whole brain, cortex, and hippocampus were derived from normalized [Gd‐DOTA] time–concentration curves, following established protocols.^27^ To evaluate global glymphatic function, we quantified whole‐brain tracer kinetics by calculating the wash‐in rate,^28^, ^31^ washout rate,^28^, ^31^ and Gd‐DOTA retention ratio^26^ at 300 min after infusion based on the normalized (Gd‐DOTA) time–concentration curves derived from the segmented brain parenchyma. The concentration of Gd‐DOTA at each voxel was converted from signal intensity using T_1_ maps obtained from VFA‐SPGR acquisitions and subsequently averaged across all brain voxels to yield a brain‐wide TSC.

The wash‐in rate was defined as the slope of signal enhancement from baseline to peak and calculated as: $Wash_{in}\;rate=\frac{Normalized\;Gd_{max}-Normalized\;Gd_{pre}}{TTP}$), where $Gd_{\max}$ represents the peak normalized concentration and $Gd_{\mathrm{pre}}$ the baseline (pre‐injection) concentration, and *TTP* denotes the time to peak. The washout rate reflects the rate of tracer clearance from brain tissue and was calculated from the linear slope between 90% and 10% of the peak signal intensity: $Wash_{out}\;rate=\frac{0.9\cdot \;Gd_{max}-0.1\cdot Gd_{max}}{t_{90\%}-t_{10\%}}$, where $t_{90\%}$ and $t_{10\%}$ correspond to the time points at which the normalized signal decayed to 90% and 10% of the peak, respectively. The retention ratio at 300 min was used as a semi‐quantitative index of long‐term tracer retention in the brain, calculated as follows: $Retention\;\;ratio_{300\;min}=\frac{Gd_{300\;min}-Gd_{pre}}{Gd_{max}-Gd_{pre}}\;\times 100\%$. This metric reflects the proportion of the initially accumulated tracer that remained in the brain parenchyma at the 300‐min time point, thus serving as an indirect index of glymphatic clearance efficiency.

### 2.7 Measurement of glymphatic efflux

Glymphatic efflux was assessed using a previously established protocol for monitoring parenchymal tracer clearance into the systemic circulation through real‐time intravascular fluorescence imaging. Briefly, the mice were stereotaxically implanted with a guide cannula (26‐G; C315G SPC; 4.5 mm below pedestal; PlasticsOne; Roanoke, VA, USA) targeting the right striatum (coordinates from bregma: anteroposterior, +0.65 mm; mediolateral, −2.0 mm; dorsoventral, −2.9 mm). The guide cannula was sealed with a dummy cannula (33‐G; C315DC/SP; 4.5‐mm projection). The mice were allowed to recover for 24 h. On the day of the measurement, the mice were anesthetized with 2% isoflurane, and the left femoral vein was surgically exposed to facilitate intravascular imaging. Imaging was performed using a fluorescence stereomicroscope (Echo Revolve, Echo, San Diego, CA, USA), with the excitation wavelength set to 488 nm. A total of 1 µL of 4% (w/v) Direct Blue 53 (also known as Evans Blue; E2129, Sigma‐Aldrich, USA) in phosphate‐buffered saline (PBS) was infused into the right striatum at a rate of 0.2 µL/min using a 26s‐G syringe (No. HAM80075; Hamilton). Real‐time fluorescence images of the femoral vein lumen were captured every 15 min over a period of 3 h. Tissue hydration at the surgical site was maintained with sterile 0.9% saline throughout the imaging session. The images were analyzed using ImageJ (National Institutes of Health, Bethesda, MD, USA). The region of interest (diameter = 0.1 mm) was positioned over the femoral vein on the basis of the overlaid fluorescence signal at 488 nm. Background fluorescence was corrected by subtracting the signal from a second region of interest of identical size placed approximately 1 mm from the vessel.

### 2.8 Quantification of AQP4 polarization

To quantify AQP4 polarization, 50‐µm segments centered on blood vessels (identified using vascular‐shaped AQP4 localization) were analyzed using the line‐plot analysis tool in ImageJ. The selected vessels measured approximately 7 µm in width (ranging from 6 to 8 µm). Fluorescence signals from CD31 (marking blood vessels) and glial fibrillary acidic protein (GFAP) (marking astrocytic endfeet) were used to delineate the perivascular space. The average AQP4 intensity within the perivascular space was calculated to represent the distribution of AQP4 specifically localized at the vascular endfeet. Additionally, the average AQP4 intensity in the extraperivascular space, defined as the region extending 15 µm from the boundary of the identified perivascular space, was calculated to determine the distribution of AQP4 outside the perivascular space. The ratio of perivascular AQP4 intensity to extraperivascular AQP4 intensity was calculated to generate an arbitrary value representing the AQP4 polarization index. A higher AQP4 polarization index value indicated a greater proportion of AQP4 immunoreactivity restricted to perivascular regions, whereas a lower AQP4 polarization index value indicated a more uniform distribution of AQP4 between the perivascular endfeet and astrocytic soma.

### 2.9 Positron emission tomography

A 30‐min transmission scan (involving a germanium‐68 pin source with an activity of 18.5 MBq) was conducted to correct for attenuation in the imaging process. A catheter was inserted into the tail vein to inject Florbetaben F‐18 (Neuraceq), which was synthesized using a nucleophilic substitution reaction and an automated Tracerlab FX–FN cartridge synthesizer (General Electric Co., Boston, MA, USA) by Vertas Biomedical, Taipei, Taiwan. The mice were placed on a small‐animal positron emission tomography (PET) scanner (SuperArgus, SEDECAL, Madrid, Spain) with their heads immobilized. The scanner was set to three‐dimensional acquisition mode. Axial resolution was set to 0.9 mm at the center of the field of view. Acquisition commenced immediately following radiotracer injection. The obtained images were reconstructed using filtered back‐projection with a Shepp filter (cutoff frequency: 0.35 cycles/pixel) into a 128 × 128 × 47 matrix with a voxel size of 1.7 × 1.7 × 2.4 mm^3^, corrected for attenuation, and realigned to generate sagittal and coronal views. The radiotracer was administered at a dose of 0.37 to 0.56 MBq/g of body weight in 0.1 mL of physiological saline. All data were reconstructed in user‐defined timeframes. Region‐of‐interest analysis was performed in AsiProVM (CTI Concorde Microsystems). Regions of interest associated with different brain regions were drawn on all coronal brain images, guided by stereotactic coordinates. Decay‐corrected time–activity curves were constructed and expressed as standardized uptake value ratios and normalized to body weight to account for variations in mouse weight and doses.

### 2.10 Tissue collection and brain fixation

The mice were anesthetized with isoflurane and euthanized at 3 months after BCCAO for the surgery groups or at 8 months of age for the sham groups. For histological analysis, the mice were first perfused with PBS and then with 4% paraformaldehyde to preserve brain tissue. Brains were extracted and immersed in a 30% sucrose solution until they sank and then sectioned into 50‐µm slices using a freezing microtome. Specific brain regions, including the cortex and hippocampus, were isolated separately for Western blotting and reverse transcription polymerase chain reaction array. Cortex tissues from the left and right hemispheres were collected as separate samples. Because of the small size of the hippocampus, tissues from both hemispheres were pooled to obtain sufficient material for analysis. Tissue samples were immediately frozen in liquid nitrogen and stored at −80°C.

### 2.11 Tissue homogenization, soluble protein extraction, and Western blotting

Mouse brain tissue was first homogenized on ice in an appropriate volume of an ice‐cold buffer (120‐mM sodium chloride, 50‐mM Tris, and protease and phosphatase inhibitors; pH 8.0). Homogenization was performed using an 18‐G needle attached to a 1‐mL syringe. The tissue fraction was passed through the needle 10 to 15 times. Then the homogenate was passed through a 27‐G needle 10 to 15 times to ensure complete tissue disruption. After homogenization, the samples were centrifuged at 14,600 × g for 30 min at 4°C. The supernatant, representing the soluble protein fraction, was carefully collected and stored at −80°C for subsequent analysis.

The pellet was resuspended in radioimmunoprecipitation lysis buffer (Millipore Sigma, Billerica, MA, USA) containing protease and phosphatase inhibitors. Next, a bicinchoninic acid assay was performed. Equal amounts of protein (30 µg per lane) were resolved through sodium dodecyl sulfate polyacrylamide gel electrophoresis, and the protein bands were transferred onto polyvinylidene fluoride membranes. The membranes were blocked with a blocking buffer (BlockPRO; Catalog No. BF01; Visual Protein) for 30 min at room temperature. After washing, the membranes were incubated overnight at 4°C with primary antibodies specific to phosphorylated tau 217 (p‐tau217; GTX135775; Gentex) or actin (Catalog No. 58169; Cell Signaling) diluted in Tris‐buffered saline with Tween 20 (TBST) containing 5% bovine serum albumin). After washing, the membranes were incubated with corresponding horseradish peroxidase–conjugated anti‐rabbit/anti‐mouse secondary antibodies (Millipore Sigma) for 1 h at room temperature. Protein bands were visualized using a chemiluminescent substrate (Catalog No. NEL113001EA; Western Lightning Ultra Chemiluminescence; PerkinElmer) and imaged using a BioSpectrum imaging system (UVP, Upland, CA, USA).

### 2.12 Immunofluorescence staining

Prefrontal cortex and hippocampal tissue sections were subjected to immunofluorescence staining. In brief, the tissue sections were blocked for 1 h in a solution of 0.2% Triton X‐100, 6% horse serum, and PBS. After washing, the sections were incubated overnight at room temperature with primary antibodies. After incubation, the sections were washed with TBST and incubated overnight at room temperature with corresponding secondary antibodies. After another wash with TBST, the stained tissue sections were mounted using UltraCruz Antifade Mounting Medium containing 4′,6‐diamidino‐2‐phenylindole (sc‐24941; Santa Cruz Biotechnology) and sealed with nail polish. The sections were then examined and scanned using either an Evos or Confocal microscope (DMI 6000B CS). The following primary antibodies were used: Aβ (GTX134510; GeneTex), ionized calcium‐binding adaptor molecule 1 (ab4674; Abcam), and CD16/32 (AF3628; Invitrogen). The secondary antibodies were as follows: CF488A, CF568, and CF633 conjugated secondary antibodies from Biotium (Hayward, CA, USA).

For p‐tau217, mouse brain tissues were fixed in 4% paraformaldehyde in PBS overnight at 4°C. After fixation, the paraffin‐embedded tissues were sectioned using a microtome into 5‐µm‐thick slices and mounted onto glass slides. Before staining, the sections were deparaffinized in NOVA Histo (BIONOVAS. LB0200). Antigen retrieval was performed by heating the sections in citrate buffer (10 mM sodium citrate; pH 6.0) at 95°C to 100°C for 15 to 20 min. After cooling to room temperature, the sections were washed with PBS and permeabilized for 1 h at room temperature with 0.3% Triton X‐100 containing 5% normal goat serum in PBS. To determine the cellular localization of p‐tau217, sections were incubated overnight at room temperature with primary antibodies against p‐tau217 (Gentex), NeuN (neuronal marker), Iba1 (microglial marker), and GFAP (astrocytic marker) diluted in blocking buffer. After washing with TBST, sections were incubated with species‐specific secondary antibodies conjugated to spectrally distinct fluorophores, selected according to the host species of each primary antibody. Following a second TBST wash, sections were mounted using UltraCruz Antifade Mounting Medium containing 4′,6‐diamidino‐2‐phenylindole (DAPI; sc‐24941, Santa Cruz Biotechnology) and sealed with nail polish. Fluorescent images were acquired using a 40× objective on a confocal laser scanning microscope (STELLARIS 8, Leica Microsystems).

### 2.13 Library preparation for Chromium Single Cell Flex Gene Expression

Samples were prepared using the Chromium Next GEM Single Cell Fixed RNA Sample Preparation Kit (10x Genomics) to generate fixed single‐cell suspensions and then stored at −80°C according to the manufacturer's protocol. After thawing, the concentration of fixed cells was determined using the Countess III FL Automated Cell Counter (Thermo Fisher Scientific), and cells were hybridized overnight with the barcoded whole transcriptome probe pairs. Gel beads‐in‐emulsion (GEMs) were generated by combining barcoded Single Cell Gel Beads, a master mix containing pooled probe‐hybridized cells, and partitioning oil onto the Chromium Next GEM Chip Q, and partitioning was performed using the Chromium X instrument. Briefly, probe ligation and extension were performed inside GEMs in a thermal cycler to generate barcoded products from hybridized probes, followed by polymerase chain reaction (PCR) amplification. Final libraries were constructed via sample index PCR and were purified using the SPRIselect Reagent Kit (Beckman Coulter). Libraries were assessed using the Qsep400 System (BiOptic Inc., Taiwan) and quantified with a Qubit Fluorometer (Thermo Fisher Scientific). Sequencing was performed on the Illumina NovaSeq X Plus platform (paired‐end 150 bp reads) by Genomics, BioSci & Tech Co., New Taipei City, Taiwan.

### 2.14 Single‐cell RNA sequencing analysis

Raw sequencing reads were processed and aligned using Cell Ranger version 8.0 (cellranger multi, 10x Genomics). Downstream analyses were performed in R version 3.6. Quality control, normalization (SCTransform), dimensionality reduction (PCA, UMAP, and t‐SNE), clustering (shared nearest‐neighbor and Louvain algorithms), and marker gene identification were conducted with Seurat version 5.1.0. Cell‐type annotation was performed using curated marker gene sets in combination with scCATCH version 2.1. Visualization of cell clustering and gene expression patterns was carried out with Seurat, and cell–cell communication analyses were conducted using CellChat version 2.1.2.

### 2.15 Adrenergic receptor blocker treatment

To restore vascular functional tone under CCH, a cocktail of adrenergic receptor (AdR) blocker was administered to WT‐BCCAO and AD‐BCCAO mice. The AdR blocker mixture consisted of prazosin hydrochloride (6 mg/kg; Sigma–Aldrich), propranolol hydrochloride (6 mg/kg; Tocris Bioscience), and atipamezole hydrochloride (0.6 mg/kg; Tocris Bioscience), prepared in 0.1% DMSO in sterile saline. The cocktail was administered via intraperitoneal injection every 2 days, starting 1 month after BCCAO and continuing until the assessment of glymphatic efflux (Direct Blue 53 experiment), vascular pulsatility and resistance indices, immunofluorescence staining for VEGF localization, and behavioral testing using the novel object recognition test. Sham groups received an equivalent volume of vehicle.

### 2.16 Statistical analysis

Data are presented as mean ± standard deviation values. Multigroup comparisons were performed using one‐ or two‐way analysis of variance, followed by Bonferroni's post hoc test. Statistical significance was set at *p* < 0.05. All statistical analyses were performed using Prism (version 9; GraphPad).

## 3 RESULTS

### 3.1 BCCAO led to cognitive decline in both WT and AD mice

**FIGURE 1:**
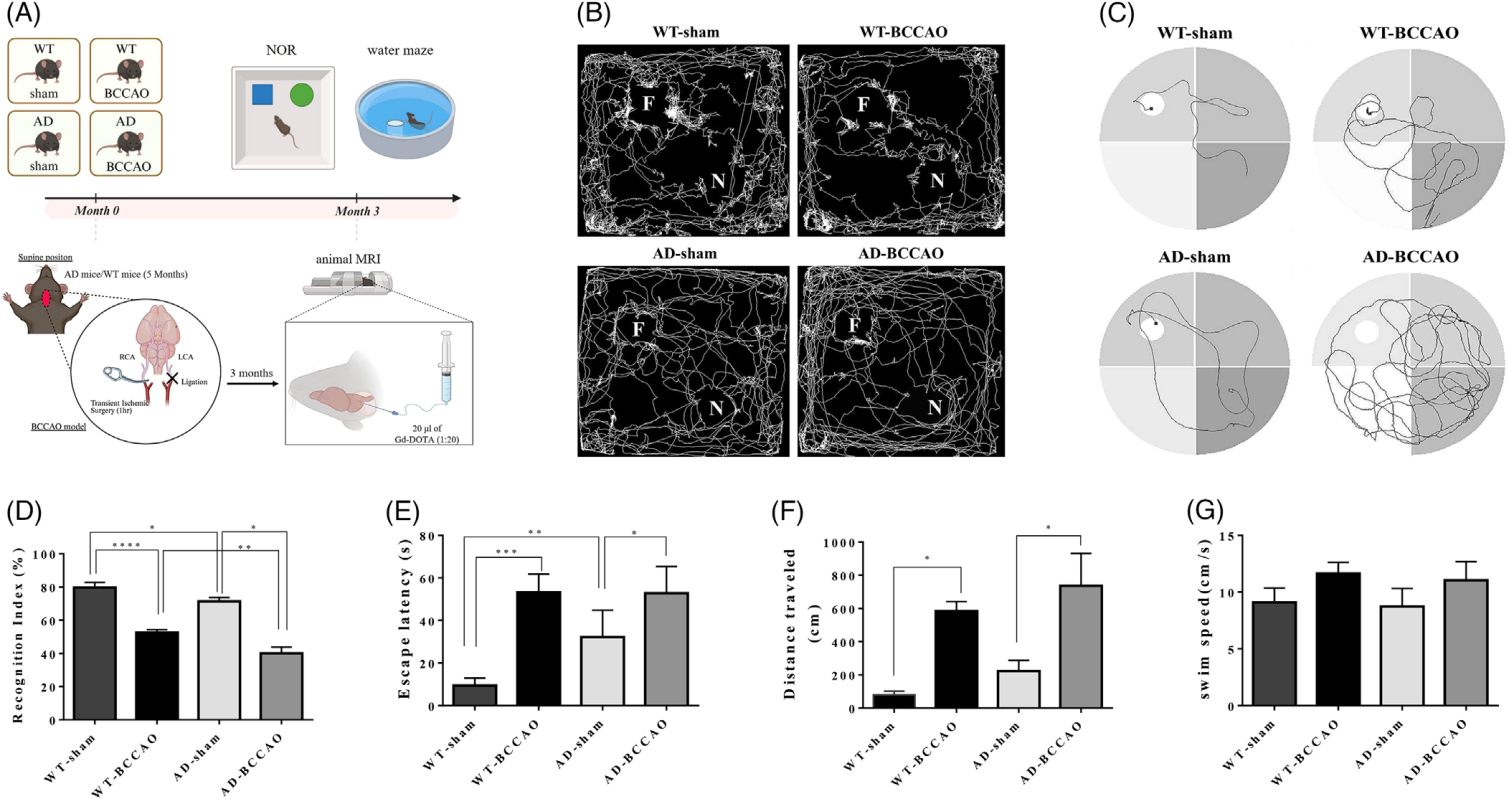
Effect of BCCAO on memory and learning abilities. (A) Overview of experimental design. (B) Performance of experimental groups in novel object recognition test. (C) Representative swim paths of experimental groups in Morris water maze test. (D) In novel object recognition test, recognition index values were significantly lower in AD‐BCCAO group than in AD‐sham, WT‐BCCAO, and WT‐sham groups. (E) In Morris water maze test, both AD‐BCCAO and WT‐BCCAO groups exhibited significantly longer escape latencies in finding the hidden platform than did the AD‐sham and WT‐sham groups. (F) Similarly, the AD‐BCCAO and WT‐BCCAO groups traveled significantly greater distances to reach the platform than did the AD‐sham and WT‐sham groups. (G) No significant differences in swim speed were observed among the four experimental groups. *p < 0.05, **p < 0.01, ***p < 0.001, ****p < 0.0001. AD, Alzheimer's disease; BCCAO, bilateral common carotid artery occlusion; WT, wild type.

To confirm our hypothesis that CCH impaired cognitive function, we conducted novel object recognition and Morris water maze tests, thereby evaluating memory and learning abilities in four experimental groups at 3 months after surgery (either BCCAO or sham) (Figure 1A). In the novel object recognition test, recognition index values were significantly lower in the BCCAO groups than in the sham groups. The AD‐BCCAO group scored the lowest, followed by the WT‐BCCAO, AD‐sham, and WT‐sham groups (Figure 1B,D). Similar results were obtained in the Morris water maze test. The BCCAO groups took longer and traveled greater distances than did the sham groups (Figure 1C,E,F). No significant between‐group difference was noted in swim speed, suggesting that motor ability did not confound the assessment of cognitive performance (Figure 1G). AD mice exhibited poorer cognitive function than did WT mice, and BCCAO worsened this decline. These findings indicate that CCH exacerbates cognitive impairment, irrespective of the presence of neurodegenerative pathology.

### 3.2 BCCAO‐induced changes in CBF, pulsatility, and resistance confirmed CCH

BCCAO significantly reduced CBF (at both the 1‐ and 3‐month follow‐ups) in the WT and AD groups (Figure S1A). The AD group had lower CBF (both before and after surgery) than did the WT group, which highlighted the potential synergistic effect of BCCAO and AD pathology on cerebral perfusion. Pulsatility and resistance index values significantly increased after BCCAO and remained elevated over the 3‐month follow‐up period, indicating sustained changes in cerebral hemodynamics (Figure S1B,C).

### 3.3 Postoperative impairment of glymphatic function was associated with reduced wash‐in and washout kinetics

**FIGURE 2:**
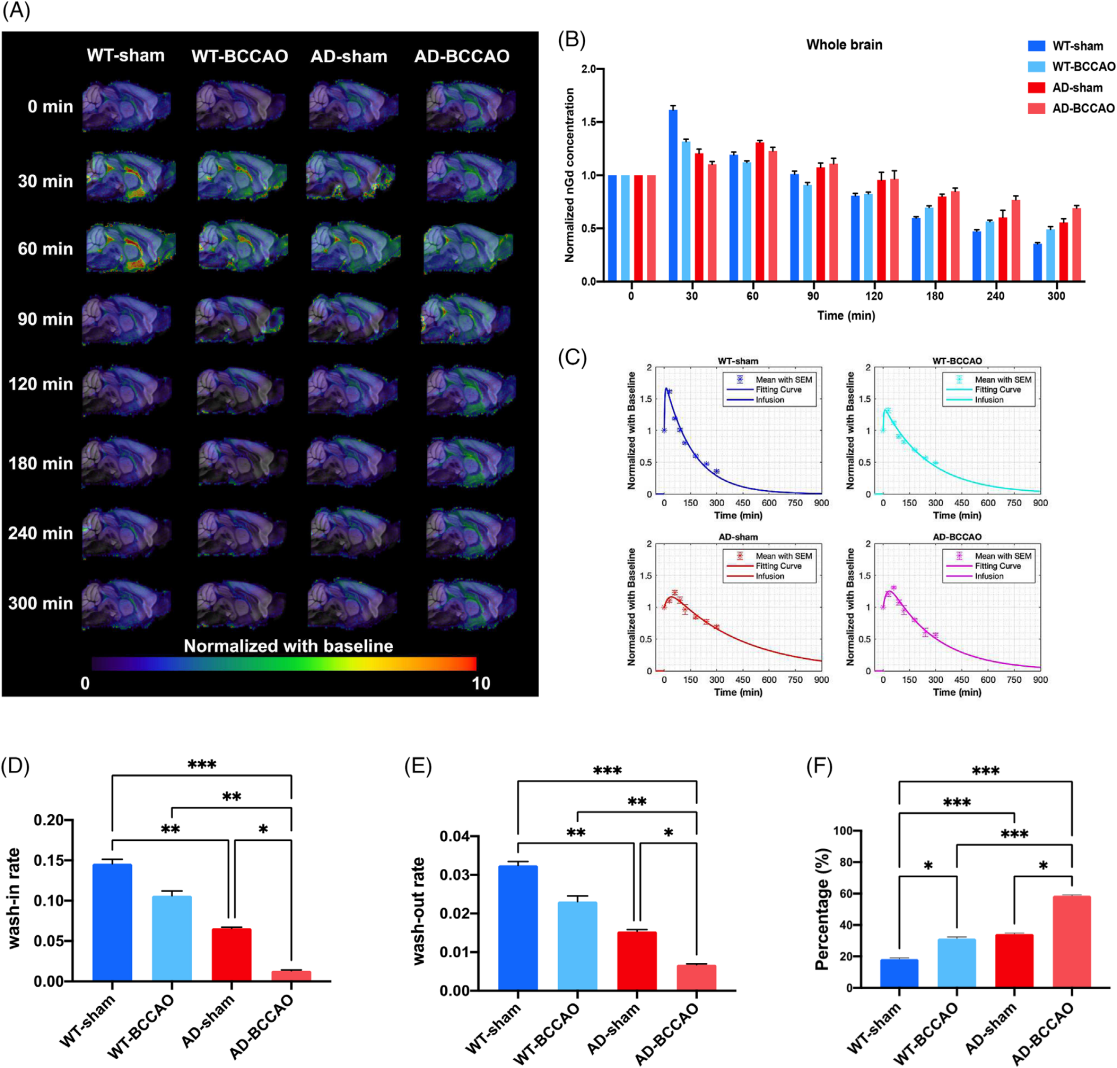
Glymphatic function. (A) Gadobutrol concentrations in the whole brain at different time points. (B) Gadobutrol concentrations in the whole brain at baseline and 30, 60, 90, 120, 180, 240, and 300 min after injection. In the WT‐sham and WT‐BCCAO groups, gadobutrol reached the highest concentration at 30 min after injection. In the AD‐sham and AD‐BCCAO groups, gadobutrol reached the highest concentration at 60 min after injection. (C) Curves depicting changes in gadobutrol concentration with time: The WT‐sham group exhibited the sharpest slopes of increase and decrease, followed by the WT‐BCCAO, AD‐sham, and AD‐BCCAO groups. Rates of (D) wash‐in and (E) washout and (F) the proportion of gadobutrol in the brain at 300 min; the findings indicated that BCCAO and AD impaired glymphatic function. *p < 0.05, **p < 0.01, ***p < 0.001. AD, Alzheimer's disease; BCCAO, bilateral common carotid artery occlusion; WT, wild type.

Gd‐DOTA concentration (in whole brain without ventricles) reached its peak at 30 min after injection in the WT‐BCCAO and WT‐sham groups and 60 min after injection in the AD‐BCCAO and AD‐sham groups (Figure 2A–C; Table S1). The WT‐sham group exhibited the highest wash‐in and washout rates, followed by the WT‐BCCAO, AD‐sham, and AD‐BCCAO groups (Figure 2D,E; Tables S2 and S3). At 300 min after injection, the AD‐BCCAO group retained the highest proportion of Gd‐DOTA in the brain, followed by AD‐sham, WT‐BCCAO, and WT‐sham groups (Figure 2F; Table S4). Gd‐DOTA concentrations in the cortex and hippocampus matched those in the whole brain without ventricles (Figures S2 and S3; Tables S5 and S6). Together, the findings indicated that the AD group had poorer glymphatic function than did the WT group and that BCCAO impaired glymphatic clearance and wash‐in and washout kinetics.

### 3.4 BCCAO impaired glymphatic efflux

**FIGURE 3:**
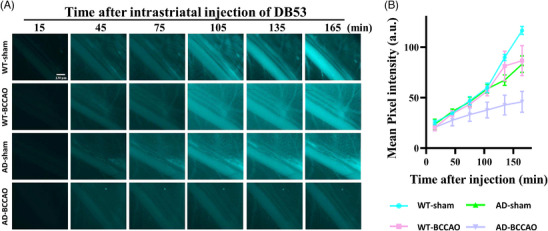
Glymphatic efflux measured through real‐time femoral vein imaging with Direct Blue 53 injection into brain parenchyma. (A) Fluorescence intensity in femoral vein. (B) Fluorescence intensity was the lowest in the AD‐BCCAO group; it was lower in the WT‐BCCAO and AD‐sham groups than in the WT‐sham group. These findings indicated that both BCCAO and AD impaired glymphatic efflux. AD, Alzheimer's disease; BCCAO, bilateral common carotid artery occlusion; WT, wild type.

We further investigated glymphatic clearance by monitoring glymphatic efflux in real time. For this, the femoral vein was imaged following the injection of the fluorescent tracer Direct Blue 53 into the brain parenchyma. Fluorescence intensity in the femoral vein was the lowest in the AD‐BCCAO group. The WT‐BCCAO and AD‐sham groups exhibited lower intensity levels than did the WT‐sham group (Figure 3A,B). The findings indicated that both BCCAO and AD impaired glymphatic efflux, with BCCAO causing further decline. This aligns with our previous results suggesting that BCCAO impairs washout kinetics.

### 3.5 BCCAO intensified AQP4 depolarization

**FIGURE 4:**
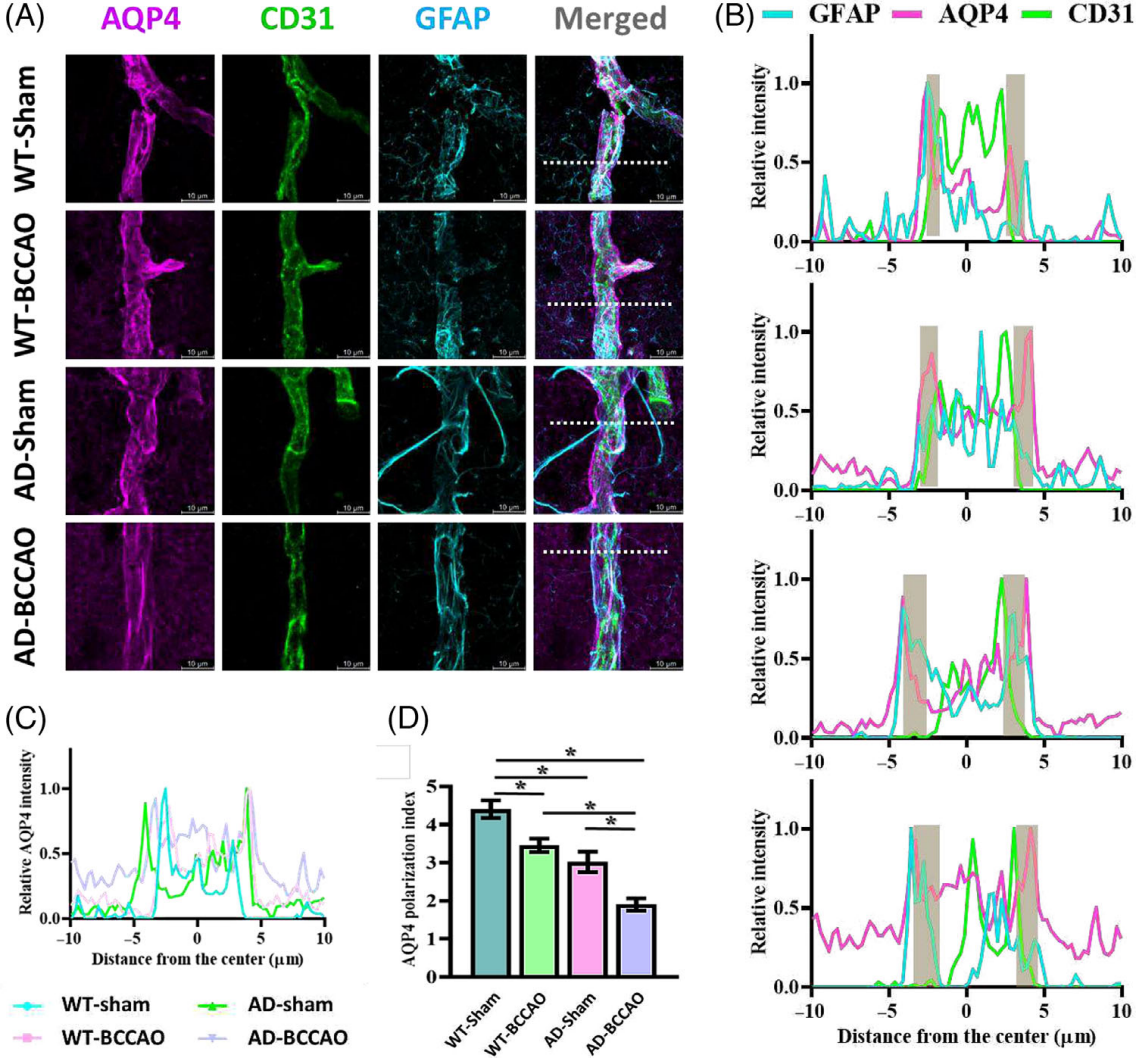
BCCAO‐induced changes in AQP4 polarization. (A) Immunofluorescence staining results of experimental groups: AQP4, magenta; CD31, green; and GFAP, cyan. Dashed lines in merged images indicate regions used for quantifying AQP4 polarization. (B) Line‐plot analyses of relative fluorescence intensity for signals shown in (A). Yellow highlights indicate perivascular space. (C) Merged image depicting relative intensity of AQP4 distribution across cross‐section of a blood vessel. (D) Quantitative analysis of AQP4 polarization in experimental groups. *p < 0.05. AQP4, aquaporin‐4; CD31, platelet endothelial cell adhesion molecule‐1 (PECAM‐1); GFAP, glial fibrillary acidic protein; BCCAO, bilateral common carotid artery occlusion.

AQP4 plays a key role in regulating the glymphatic system. Therefore, we investigated the spatial distributions of AQP4, CD31 (endothelial marker of blood vessel), and GFAP (astrocytic endfeet) and evaluated the integrity and organization of the perivascular astrocytic network (Figure 4A). In the WT‐sham group, the immunoreactivity of AQP4 was predominantly localized to the perivascular endfeet, as evidenced by strong AQP4 signal intensity along CD31‐positive (CD31^+^) blood vessels and colocalization with GFAP‐labeled astrocytic processes. Distinct spatial differences were observed in the distribution of AQP4 (Figure 4B). The localization of AQP4 at the vascular endfeet was lower in the WT‐BCCAO and AD‐sham groups than in the WT‐sham group. Merged images of cross‐sectional fluorescence intensities visually confirmed the altered AQP4 distribution (Figure 4C). Quantitative analysis of the AQP4 polarization index (Figure 4D) revealed that BCCAO led to a modest reduction in AQP4 polarization, and AD also caused a slight reduction in AQP4 polarization. The reduction in AQP4 polarization in the AD‐BCCAO group was significantly larger than those in the AD‐sham and WT‐BCCAO groups, highlighting the potential synergistic effect of ischemia and neurodegeneration on AQP4 distribution. These findings indicate that the polarization of AQP4 is disrupted under conditions of CCH and AD, with the combined insults markedly impairing the localization of this protein in perivascular regions. Given the pivotal role of polarized AQP4 in glymphatic fluid transport, this disruption likely leads to glymphatic dysfunction.

### 3.6 BCCAO increased Aβ accumulation and stasis

**FIGURE 5:**
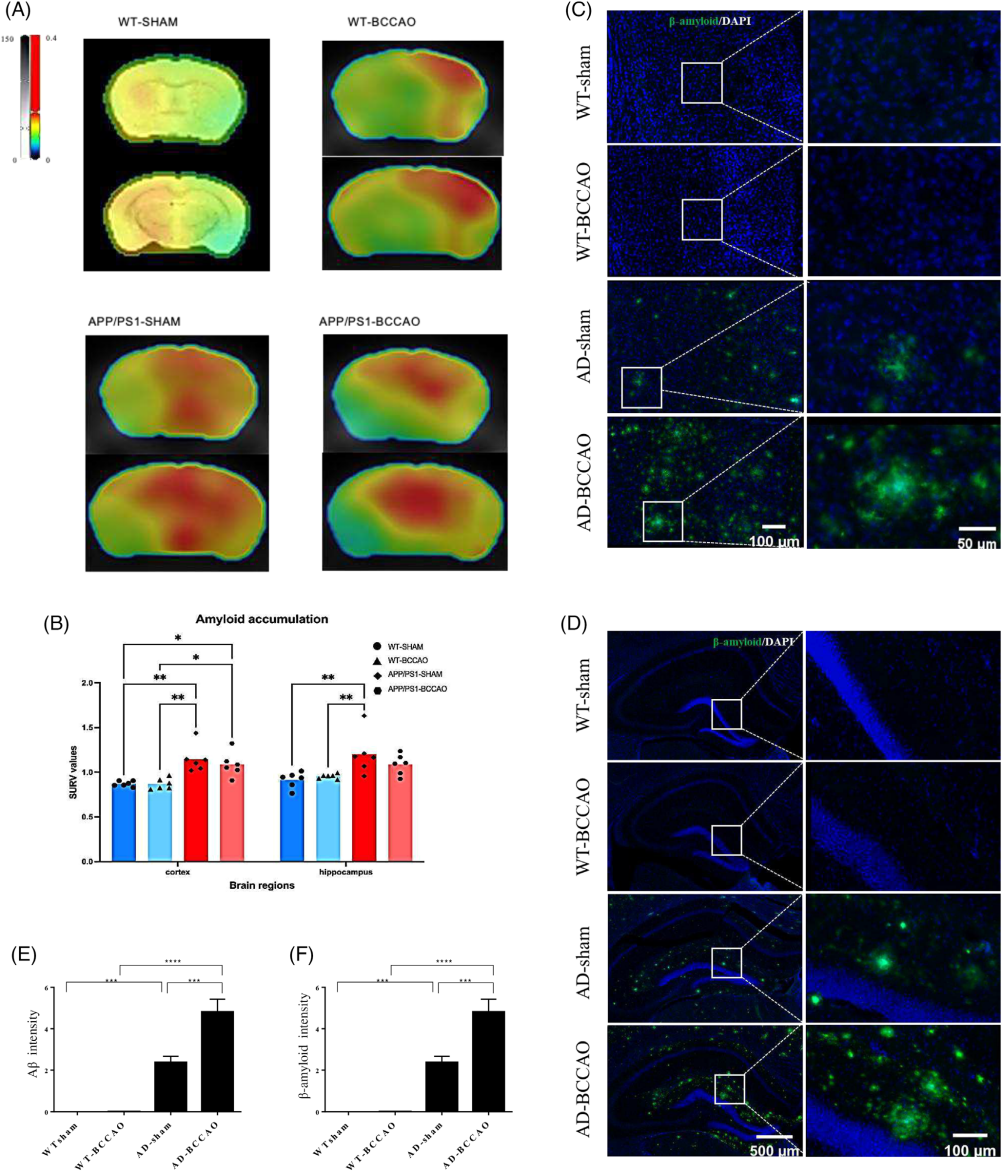
Accumulation of Aβ in the brain. (A) PET scan for Aβ. (B) Quantification of PET results: standardized uptake value ratios for two brain regions. (C) Immunofluorescence of Aβ markers in cortex. (D) Immunofluorescence of Aβ markers in dentate gyrus. (E) Immunofluorescence in cortex indicated accumulation of Aβ in both AD‐BCCAO and AD groups, with greater intensity in AD‐BCCAO group than AD group. (F) Immunofluorescence in dentate gyrus indicated accumulation of Aβ in both AD‐BCCAO and AD groups, with greater intensity in AD‐BCCAO group than AD group. *p < 0.05, **p < 0.01, ***p < 0.001, ****p < 0.0001. Aβ, amyloid beta; AD, Alzheimer's disease; BCCAO, bilateral common carotid artery occlusion; WT, wild type; PET, positron emission tomography.

To evaluate the effect of CCH on Aβ accumulation, we performed amyloid PET imaging and immunofluorescence staining to assess Aβ burden. PET images revealed greater Aβ accumulation in the AD group than in the WT group (Figure 5A). Post hoc analysis indicated no significant differences in standardized uptake value ratios between the AD‐BCCAO and AD‐sham groups, but the AD group had significantly higher standardized uptake value ratios than did the WT group (Figure 5B). Immunofluorescence staining yielded similar results (i.e., greater Aβ accumulation in the AD group than in the WT group in both the cortex and dentate gyrus; Figure 5C,D). Notably, unlike PET imaging, immunofluorescence staining revealed greater Aβ accumulation in the cortex and dentate gyrus in the AD‐BCCAO group than in the AD‐sham group, suggesting that BCCAO exacerbates Aβ accumulation and stasis in these regions (Figure 5E,F).

### 3.7 BCCAO increased p‐tau217 accumulation and stasis

**FIGURE 6:**
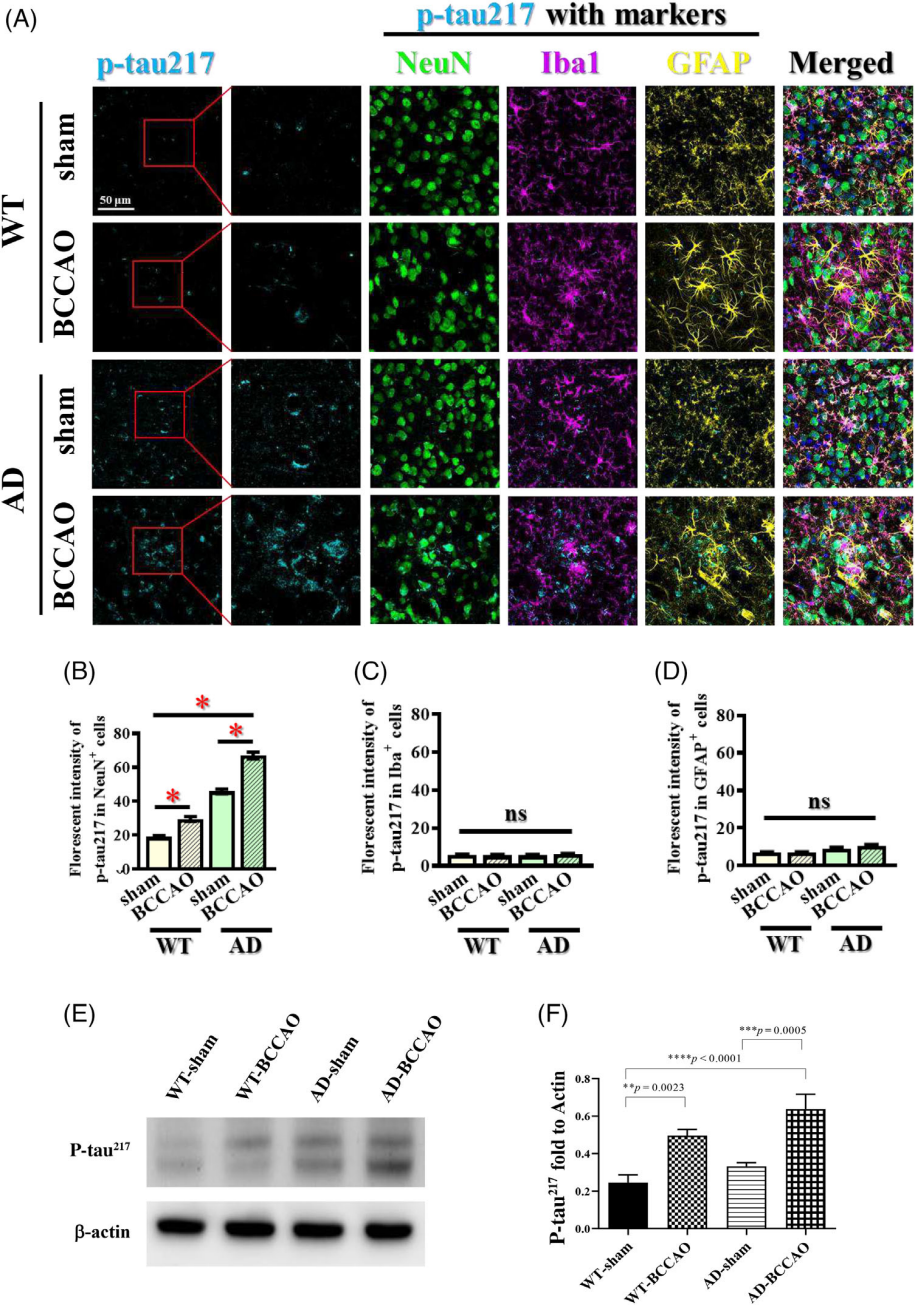
Cellular localization and cell‐type‐specific distribution of p‐tau217 in cortex. (A) Representative immunofluorescence images showing colocalization of p‐tau217 with cell‐type‐specific markers: NeuN (neurons), Iba1 (microglia), and GFAP (astrocytes) across all four experimental groups. P‐tau217 signals predominantly colocalize with NeuN‐positive neurons, with minimal overlap observed with microglia or astrocytes under all conditions. (B–D) Quantification of p‐tau217 fluorescence intensity in neurons (B), microglia (C), and astrocytes (D) across the four experimental groups. (E) Representative Western blot analysis showing cortical p‐tau217 levels in all four experimental groups. (F) Quantification of p‐tau217 protein levels demonstrates increased accumulation in BCCAO groups compared with corresponding sham groups. AD, Alzheimer's disease; BCCAO, bilateral common carotid artery occlusion; GFAP, glial fibrillary acidic protein; Iba1, ionized calcium‐binding adapter molecule 1; NeuN, neuronal nuclei; WT, wild type.

We next evaluated the effect of BCCAO on p‐tau217 accumulation. BCCAO significantly increased p‐tau217 levels in both WT and AD mice, with the increase predominantly localized to cortical neurons but not observed in microglia or astrocytes (Figure 6A–D). These observations were corroborated by Western blot analysis, which showed significantly greater p‐tau217 accumulation in BCCAO groups compared with sham controls (Figure 6E). Quantitative analysis of p‐tau217 normalized to beta‐actin revealed that the AD‐BCCAO and WT‐BCCAO groups had higher soluble p‐tau217 levels than did the AD‐sham and WT‐sham groups, indicating that BCCAO increases p‐tau217 accumulation (Figure 6F). These findings suggest that CCH exacerbates tau pathology in both normal and AD contexts, accelerating neurodegeneration through increased neuronal tau phosphorylation.

### 3.8 BCCAO suppresses gliovascular VEGF signaling and network crosstalk

To interrogate cell‐type‐specific mechanisms in WT and AD mice under BCCAO, we performed scRNA‐seq and integrated four datasets – WT‐sham (9343 cells), WT‐BCCAO (13,517), AD‐sham (10,831), and AD‐BCCAO (11,991) groups – with cell types annotated using canonical markers (Figure S4A,B). Overall cellular composition was broadly preserved across experimental conditions (Figure S4C). In contrast, CellChat connectomics revealed a global contraction of communication networks after BCCAO (Figure S4D). Network summaries showed significant decreases in both the number and aggregate strength of inferred ligand–receptor interactions, with the largest losses in the AD‐BCCAO group (Figure S4E). These findings suggest that BCCAO diminishes cellular cross talk within the brain microenvironment, particularly in the context of AD pathology.

**FIGURE 7:**
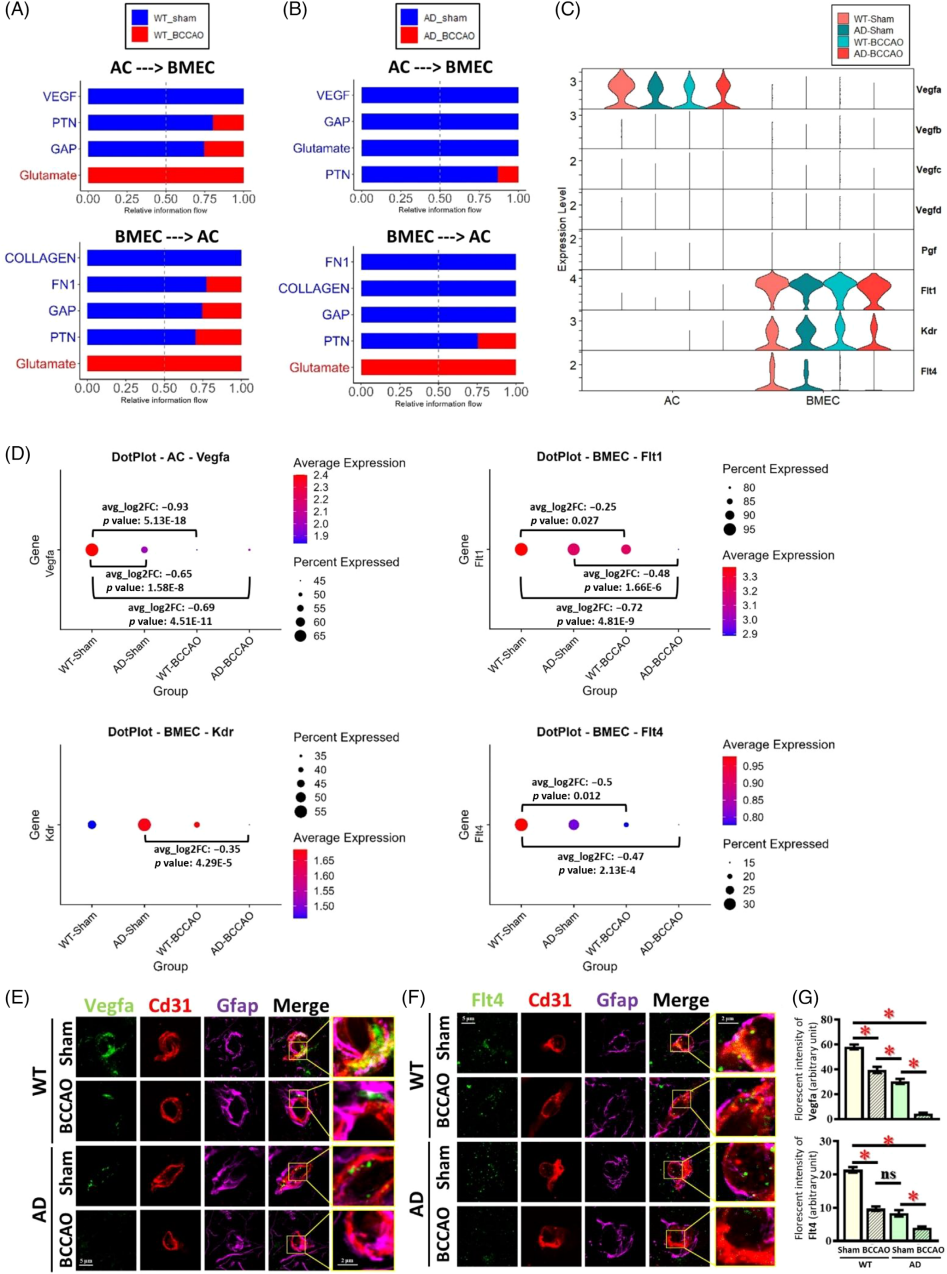
Astrocyte‐endothelial VEGF signaling is remodeled by BCCAO. (A) CellChat analysis of communication between AC and BMEC. Bars represent relative information flow (aggregate communication probability) for key signaling pathways from donor to receiver (AC to BMEC and BMEC to AC) in WT group and (B) AD group, comparing sham (blue) and BCCAO (red) conditions. VEGF, PTN, GAP, glutamate, FN1, and collagen pathways are prominently altered after BCCAO. (C) Violin plots showing expression of VEGF‐axis ligands (Vegfa, Vegfb, Vegfc, Vegfd, Pgf) and receptors (Flt1, Kdr, Flt4) in AC and BMEC across the four conditions. (D) Differential‐expression DotPlots for Vegfa in AC and Flt1, Kdr, and Flt4 in BMEC. Dot size indicates percentage of expressing cells; color indicates average scaled expression. Brackets indicate tested contrasts with average log2 fold change and Wilcoxon *p* values, showing BCCAO‐associated downregulation of astrocytic Vegfa and endothelial VEGF receptors, with stronger effects in AD‐BCCAO group. (E) Representative images showing fluorescent signals of Vegfa (green), Cd31 (red), and Gfap (magenta) in different groups of mice. (F) Representative images showing fluorescent signals of Flt4 (green), Cd31 (red), and Gfap (magenta) in different groups of mice. (G) Quantitative analysis of fluorescence intensity for Vegfa (upper panel) and Flt4 (lower panel). AC, astrocyte; AD, Alzheimer's disease; BCCAO, bilateral common carotid artery occlusion; BMEC, brain microvascular endothelial cell; Cd31, platelet endothelial cell adhesion molecule‐1 (PECAM‐1); FN1, fibronectin 1; GAP, gap junction; Gfap, glial fibrillary acidic protein; PTN, pleiotrophin; VEGF, vascular endothelial growth factor; WT, wild type.

Given the central role of gliovascular coupling in glymphatic function, we next focused on astrocyte (AC)–brain microvascular endothelial cell (BMEC) signaling. Information flow analysis revealed a redistribution of AC ↔ BMEC pathways after BCCAO (Figure 7A,B). Several astrocyte‐to‐endothelial (AC→BMEC) axes – including VEGF, pleiotrophin (PTN), and gap junction (GAP)‐associated signaling – exhibited reduced influence, with similar trends observed in BMEC→AC signaling. These alterations were evident in WT group and were more pronounced in the AD group. Gene‐level analyses supported these pathway‐level findings: Vegfa expression was consistently downregulated in astrocytes, while endothelial receptors Flt1, Kdr, and Flt4 were reduced in BMEC following BCCAO in both WT and AD group (Figure 7C,D).

To validate the impact of BCCAO and AD pathology on Vegfa expression along the glymphatic system, we examined the colocalization of Vegfa with Gfap‐positive astrocytic endfeet surrounding blood vessels using immunofluorescence staining. The results showed robust colocalization of Vegfa with Gfap‐positive astrocytic processes in the WT‐sham group (Figure 7E). In contrast, mice subjected to BCCAO or exhibiting AD pathology showed a marked reduction in Vegfa immunoreactivity along Gfap‐positive endfeet, indicating a disruption of Vegfa distribution in perivascular astrocytes. Notably, the combination of BCCAO and AD pathology resulted in a further reduction in Vegfa levels compared to either condition alone, suggesting an additive or synergistic effect of vascular and neurodegenerative insults on Vegfa expression (Figure 7E,G). In parallel, we assessed the expression of Flt4 within the glymphatic system by analyzing its colocalization with Cd31‐positive endothelial cells. Similar to Vegfa, Flt4 displayed strong colocalization with Cd31, consistent with its endothelial enrichment in WT‐sham group (Figure 7F). Either BCCAO or AD pathology decreased Flt4 expression, with the most pronounced decrease observed in the combined condition (Figure 7F,G).

To disentangle potential structural confounds from alterations in signaling, we first quantified astrocyte–vascular coverage by assessing the association of GFAP‐positive (GFAP^+^) astrocytic processes with CD31^+^ endothelial vessels, which revealed only a modest reduction in astrocyte–vessel coverage under CCH/Aβ conditions (Figure S5A–F). Importantly, this limited degree of astrocytic retraction did not result in complete loss of astrocyte–endothelial contact, indicating that gross structural disengagement alone is unlikely to account for the observed suppression of VEGF signaling. To further minimize the influence of astrocyte–vascular coverage on ligand–receptor readouts, we performed normalization‐based quantitative analyses at the level of individual cell types, quantifying VEGF intensity normalized to GFAP^+^ astrocytic area (Figure S5G) and, independently, Flt4 intensity normalized to individual CD31^+^ endothelial cells (Figure S5H). These analyses consistently demonstrated reduced astrocytic VEGF expression and diminished endothelial VEGF receptor signaling capacity, independent of changes in astrocyte–vessel apposition.

Pathway enrichment analysis of differentially expressed genes revealed substantial remodeling of signaling programs involved in vascular structure and motility. Key pathways included the Rho GTPase cycle, integrin‐ and actin‐cytoskeleton regulation, and signaling by VEGF and G protein–coupled receptors (GPCRs), along with MAPK6/4, TP53‐metabolic, PTEN, and KEAP1‐NRF2 signaling pathways (Figure S6A). A curated Rho‐cycle gene panel demonstrated broad downregulation of multiple Rho GTPase regulators in BCCAO versus sham in conditions across both WT and AD groups (Figure S6B). Collectively, these findings indicate that BCCAO disrupts core endothelial cytoskeletal regulation while redirecting gliovascular communication away from VEGF‐dependent mechanisms toward extracellular matrix (ECM) and neurotransmitter‐related signaling pathways, with these effects being more pronounced in the AD context.

### 3.9 AdR blocker treatment restores glymphatic function, vascular dynamics, and cognitive performance under CCH

**FIGURE 8:**
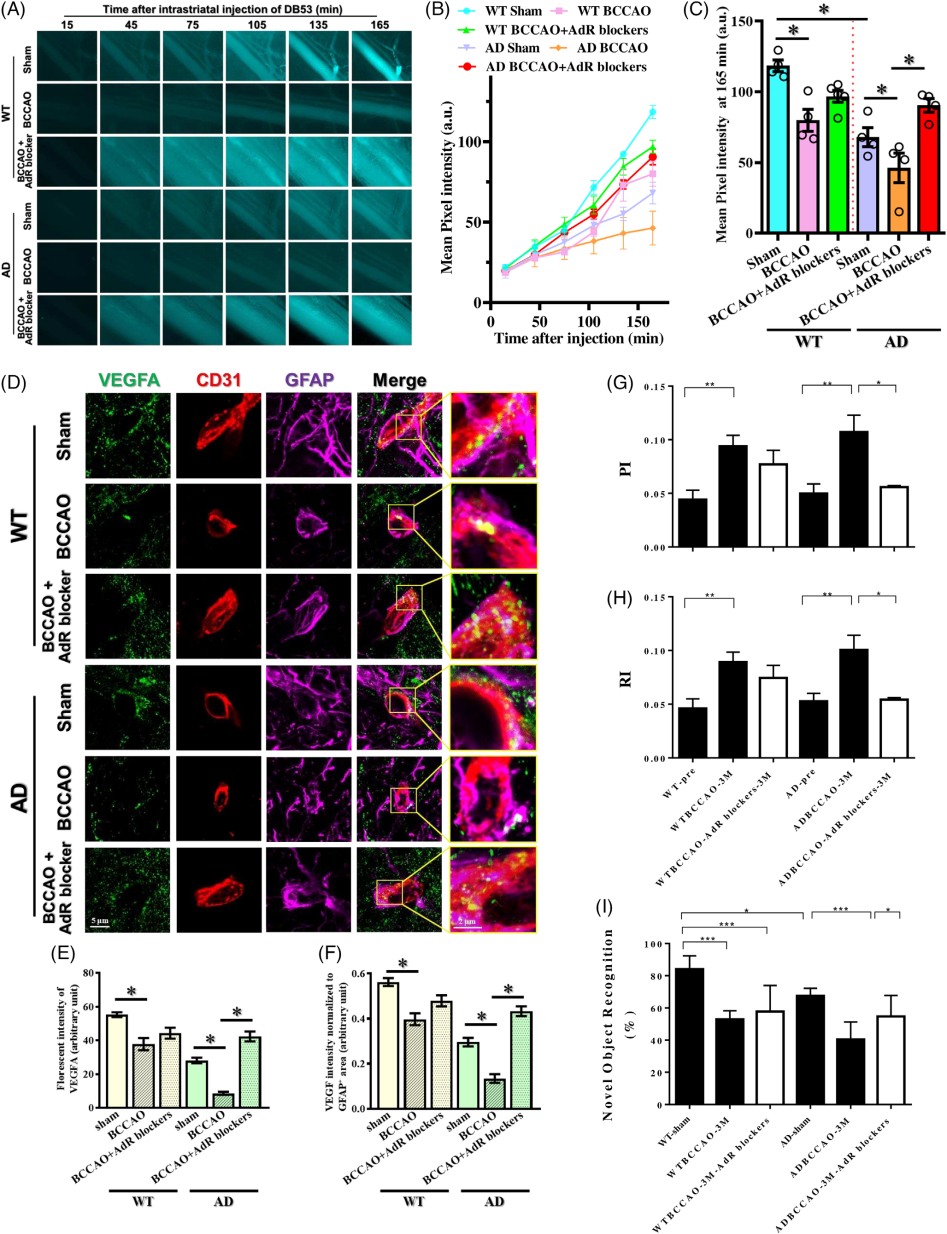
AdR blocker treatment restores glymphatic function and vascular dynamics. (A) Representative time‐course images of Direct Blue 53 fluorescence detected in femoral vein following intracerebral tracer injection in WT‐sham, WT‐BCCAO, WT‐BCCAO+AdR blocker, AD‐sham, AD‐BCCAO, and AD‐BCCAO+AdR blocker mice. (B) Quantification of mean pixel intensity of Direct Blue 53 fluorescence in femoral vein over time after tracer injection. (C) Comparison of Direct Blue 53 fluorescence intensity at 165 min after injection across experimental groups. (D) Representative immunofluorescence images showing VEGF localization at GFAP‐positive astrocytic endfeet surrounding CD31‐positive blood vessels. (E) Quantification of VEGF fluorescence intensity in perivascular regions. (F) VEGF fluorescence intensity normalized to GFAP‐positive astrocytic area. (G) Pulsatility index measurements across experimental groups. (H) Resistance index measurements across experimental groups. (I) Novel object recognition test showing recognition index across experimental groups. Data are presented as mean ± SEM. *p < 0.05; ***p < 0.0005. AdR, adrenergic receptor blocker; AD, Alzheimer's disease; BCCAO, bilateral common carotid artery occlusion; CD31, platelet endothelial cell adhesion molecule‐1 (PECAM‐1); GFAP, glial fibrillary acidic protein; PI, pulsatility index; RI, resistance index; VEGF, vascular endothelial growth factor; WT, wild type.

To test whether restoring vascular function could reverse glymphatic impairment under CCH, mice were treated with an AdR blocker cocktail, which reduces sympathetic‐mediated vasoconstriction and thereby normalizes vascular pulsatility and resistance. Glymphatic efflux assessed using the Direct Blue 53 tracer revealed a severity‐dependent pattern: WT‐sham mice showed the highest tracer appearance in the femoral vein, AD‐BCCAO mice showed the lowest, and WT‐BCCAO and AD‐sham mice were intermediate. AdR blocker treatment markedly enhanced glymphatic efflux in both WT‐BCCAO and AD‐BCCAO mice, with the most substantial recovery observed in the AD‐BCCAO+AdR blocker group (Figure 8A–C). We next examined VEGF distribution along the gliovascular interface. In WT‐sham mice, VEGF prominently colocalized with GFAP^+^ astrocytic endfeet surrounding CD31^+^ vessels. This perivascular localization was progressively disrupted in WT‐BCCAO and AD‐sham mice and most severely reduced in AD‐BCCAO mice. AdR blocker treatment restored VEGF localization at astrocytic endfeet in both WT‐BCCAO and AD‐BCCAO mice, with pronounced recovery in the AD‐BCCAO+AdR blocker group (Figure 8D–F).

Since VEGF signaling is linked to endothelial regulation of vascular resistance, we next measured pulsatility and resistance indices. Both indices progressively increased from WT‐sham to AD‐BCCAO mice, reflecting worsening vascular dysfunction with combined pathology. AdR blocker treatment reduced pulsatility and resistance indices in WT‐BCCAO and AD‐BCCAO mice, restoring values toward sham levels (Figure 8G,H). Finally, in the novel object recognition test, AdR‐treated mice exhibited a higher recognition index than untreated BCCAO mice, indicating recovery of cognitive performance (Figure 8I). These findings demonstrate that modulation of vascular tone using AdR blocker improves glymphatic clearance, restores VEGF localization at the gliovascular interface, normalizes vascular hemodynamics, and rescues cognitive function, particularly under the combined pathology of AD and CCH.

## 4 DISCUSSION

We investigated the effects of CCH on glymphatic function and cognitive decline in WT and AD mice subjected to BCCAO, establishing models for vascular and mixed‐type dementia. CCH impaired glymphatic clearance, as was evident from decreased wash‐in and washout kinetics, with loss of AQP4 polarization particularly affecting influx. Glymphatic dysfunction led to the accumulation of Aβ and p‐tau217, ultimately causing cognitive decline. Furthermore, decreased VEGF signaling, together with functional rescue by AdR blocker treatment, supports a mechanistic link between CCH and glymphatic dysfunction.

### 4.1 CCH aggravates cognitive decline

We employed a modified BCCAO model to recapitulate key pathological features of CCH, providing a stable yet survivable long‐term reduction in CBF. Compared with stenosis‐based models, BCCAO induces a more abrupt and sustained hypoperfusion, enabling study of hippocampal vulnerability, neuronal degeneration, and long‐term cognitive decline relevant to vascular cognitive impairment.^32^, ^33^ Our findings demonstrated that CCH substantially impaired memory and learning abilities, with the most severe deficits observed in the AD‐BCCAO group, indicating a synergistic interaction between CCH and AD pathology. This finding supports clinical observations, where cerebral hypoperfusion has been associated with mild cognitive impairment and dementia, particularly when combined with neurodegenerative diseases.^6^, ^34^ Vascular risk factors, such as hypertension, hypercholesterolemia, and atherosclerosis, may contribute to cerebral hypoperfusion, thereby increasing the risks of cognitive decline.^35^, ^36^, ^37^, ^38^ Notably, swim speed was unaffected, indicating that cognitive impairments were not secondary to motor impairment.

### 4.2 Sustained cerebral hypoperfusion and hemodynamic dysregulation

BCCAO disrupts cerebral hemodynamics, as indicated by reduced CBF, altered pulsatility, and increased vascular resistance. Arterial pulsation is a primary driving force for cerebrospinal fluid (CSF) movement and proper functioning of the glymphatic system.^39^, ^40^, ^41^ Increased vascular resistance and disrupted pulsatility reflect cerebral microcirculation dysfunction and are associated with cognitive impairment.^42^, ^43^ Such disruptions in the brain's vascular autoregulatory mechanisms may further accelerate neurodegeneration.^44^, ^45^, ^46^ In our study, BCCAO exacerbated cognitive decline in both WT and AD mice. Disruption of pulsatile blood flow impairs the clearance of metabolic waste products such as Aβ, linking vascular dysfunction to neurotoxic protein accumulation. This interplay between vascular insufficiency, impaired waste clearance, and neurodegeneration highlights the multifactorial nature of cognitive decline and underscores the importance of maintaining cerebrovascular health in mitigating AD pathology.

### 4.3 Glymphatic dysfunction as a mechanism underlying cerebral pathologies

Our study observed disrupted wash‐in and washout kinetics in the WT‐BCCAO and AD groups, with the AD‐BCCAO group showing the most severe impairment. This finding aligns with prior research demonstrating that both AD and CCH impair glymphatic function,^12^, ^47^, ^48^ which can lead to the accumulation and stasis of neurotoxic waste products, thereby accelerating neurodegeneration and proteinopathy.^12^, ^13^, ^49^, ^50^ In our study, CCH induced hemodynamic changes that led to glymphatic dysfunction and toxic proteinopathy. Inefficient Aβ clearance likely contributes to pathogenesis and accelerates cognitive decline.^51^, ^52^ The accumulation and stasis of Aβ and p‐tau217 was significantly associated with impaired glymphatic clearance, supporting our hypothesis that glymphatic dysfunction would be a central pathological mechanism underlying CCH‐induced cognitive impairment.

### 4.4 AQP4 depolarization links vascular injury to glymphatic failure

The glymphatic system moves CSF from perivascular spaces into the brain parenchyma via AQP4 channels, with clearance through venous and lymphatic pathways.^53^, ^54^ Changes in AQP4 polarization impair directional CSF–interstitial fluid (ISF) exchange, thereby reducing glymphatic efficiency.^55^, ^56^ Such changes have been observed in neurodegenerative diseases like AD, where disruption of AQP4 localization reduced clearance of Aβ and tau proteins.^57^, ^58^, ^59^ In our study, both AD and CCH disrupted AQP4 polarization, impairing glymphatic influx. This finding aligns with previous studies showing that AQP4‐knockout and perivascular AQP4‐deficient models exhibit significantly reduced glymphatic tracer influx and clearance.^55^, ^60^ Consistently, loss of perivascular AQP4 localization has been shown to promote Aβ plaque formation in mouse models.^14^, ^58^, ^61^ Our study demonstrated that mice with combined AD and CCH had a more pronounced loss of AQP4 polarization, implicating vascular insufficiency as acting synergistically to drive AQP4 depolarization.

CCH induces reactive astrogliosis, characterized by changes in protein concentrations and morphological features that disrupt AQP4 polarization.^62^, ^63^ Loss of the dystrophin‐associated protein complex, which anchors AQP4 to astrocytic endfeet, may also occur under CCH conditions.^64^, ^65^ In *post mortem* brain tissue from post‐stroke individuals, the proportion of clasmatodendrocytes – a form of irreversible astrocyte injury – was significantly higher in those with dementia.^66^ Abnormal AQP4 localization in these damaged astrocytes indicated disruption of astrocytic endfeet and impaired gliovascular interactions at the blood–brain barrier. These findings were further supported by a subsequent study using a baboon model of CCH, demonstrating similar astrocyte injury, AQP4 redistribution, and blood–brain barrier leakage.^66^ Our findings are consistent with these hypotheses, showing that BCCAO impaired the perivascular astrocyte network, as significant reductions in AQP4 polarization were noted in the BCCAO groups.

### 4.5 Exacerbation of Aβ and tau pathologies in CCH

The increased Aβ burden observed in the AD‐BCCAO group supports the hypothesis that CCH‐induced glymphatic dysfunction exacerbates toxic protein accumulation. PET did not show significant differences between the AD‐sham and AD‐BCCAO groups, whereas immunofluorescence staining revealed greater Aβ accumulation in the cortex and dentate gyrus in the AD‐BCCAO group. This discrepancy may reflect the predominance of non‐fibrillar or less‐compact amyloid forms in the AD‐BCCAO group, which are less detectable by conventional PET tracers that preferentially bind to β‐sheet‐rich fibrillar Aβ plaques.^67^, ^68^ Moreover, hypoperfusion‐induced glymphatic dysfunction may promote the accumulation and stasis of soluble Aβ species that evade detection by PET but are readily identified through antibody‐based techniques.

The accumulation of p‐tau217 in the cortex was significantly greater in the BCCAO groups. Studies have implicated p‐tau217 in the pathogenesis of AD, a key marker of tau‐related neurodegeneration.^69^, ^70^ The soluble form of p‐tau217 plays a vital role in early‐stage neurodegeneration, which is believed to be more neurotoxic than their aggregated counterparts.^71^, ^72^ In our study, the soluble p‐tau217 level was elevated in AD mice and further increased following BCCAO, suggesting that both AD pathology and CCH contributed to increased p‐tau217 accumulation. Accumulation of p‐tau217 following BCCAO highlights the role of CCH in promoting tau‐related neurodegeneration, even in the absence of pre‐existing Aβ pathology.

### 4.6 VEGF suppression as a convergent mechanism linking cerebral hypoperfusion, glymphatic dysfunction, and proteinopathy

Our study identified suppression of VEGF signaling and disruption of endothelial cytoskeletal programs as convergent mechanisms through which CCH and AD pathology jointly impair gliovascular function. VEGF signaling emerged as the most consistently disrupted pathway, with astrocytic Vegfa and endothelial receptors (Flt1, Kdr, Flt4) reduced in both transcriptomic and histological analyses. Normally, astrocyte‐derived VEGF promotes endothelial survival, vascular remodeling, and barrier maintenance.^73^, ^74^ Its suppression deprives endothelial cells of trophic input and weakens gliovascular coupling. These deficits were more severe in AD, where amyloid and tau pathology further compromise astrocytic metabolism and endothelial health.^75^, ^76^ This additive suppression suggests a mechanistic explanation for why hypoperfusion accelerates cognitive decline.^77^

Physiological studies have shown that VEGF‐A modulates endothelial Ca^2^^+^ signaling and IKCa channel activity in resistance arteries, thereby regulating vascular resistance and pulsatility.^78^ This provides a mechanistic link between reduced VEGF signaling and the elevated pulsatility and resistance indices observed in our model, which are key drivers of glymphatic transport. In parallel, perivascular astrocytic endfeet constitute the anatomical substrate of glymphatic inflow, and VEGF secretion likely modulates vascular permeability and perivascular fluid dynamics.^79^, ^80^ Consistent with this framework, loss of Vegfa at astrocytic endfeet and Flt4 at endothelial surfaces in our study correlated with impaired CSF–ISF exchange, glymphatic dysfunction, and protein accumulation. In addition, enrichment analyses revealed broad downregulation of Rho GTPase and integrin–actin pathways, the central regulators of endothelial shape and motility.^81^ Their impairment likely compromises barrier plasticity and perivascular pumping, further restricting glymphatic transport. Although compensatory shifts toward ECM‐ and neurotransmitter‐related pathways were observed, these were insufficient to preserve glymphatic homeostasis.

Taken together, our findings place VEGF suppression and cytoskeletal destabilization at the critical intersection of vascular insufficiency, glymphatic dysfunction, and proteinopathy. The functional relevance of this pathway is supported by AdR blocker experiments, in which restoration of vascular tone reversed VEGF mislocalization, normalized pulsatility and resistance indices, improved glymphatic efflux, and rescued cognitive performance, particularly in the AD‐BCCAO group. These findings indicate that VEGF‐dependent gliovascular coupling is functionally linked to the hemodynamic forces that drive glymphatic transport under CCH. By restoring endothelial functional responsiveness, AdR blocker treatment re‐established the gliovascular environment required for efficient glymphatic clearance, highlighting vascular tone modulation as a potential therapeutic strategy for hypoperfusion‐associated neurodegeneration.

### 4.7 Strengths and limitations

The strength of this study lies in its comprehensive experimental design, integrating behavioral testing, advanced neuroimaging, molecular profiling, and histological analyses. Additionally, scRNA‐seq enabled identification of cell‐type‐specific transcriptional changes, revealing molecular pathways through which vascular insufficiency and astroglial dysfunction impair glymphatic system. However, the study has some limitations. First, although our findings implicate glymphatic dysfunction as a mechanistic link between CCH and cognitive impairment through hemodynamically induced AQP4 dysregulation, causality remains to be established. Studies involving targeted modulation of AQP4 expression may clarify these causal relationships. Second, cognitive decline in AD mice subjected to BCCAO might be multifactorial. Factors beyond Aβ and p‐tau217 accumulation, such as neuroinflammation, should also be investigated. Third, scRNA‐seq captures only a static snapshot of transcriptomic activity, limiting its ability to fully depict dynamic cellular interactions and temporal adaptations. Moreover, scRNA‐seq cannot directly measure protein expression or signaling events such as VEGF pathway activity that are crucial for vascular–astrocytic communication. Although we did not directly manipulate individual molecular pathways such as VEGF or Rho GTPase signaling, AdR blocker treatment functionally restored VEGF localization. Future studies using more targeted genetic or pharmacological approaches may further clarify the relative contributions of these pathways. Last, although this CCH model was validated in our previous studies, it has not been widely used and therefore requires further validation.

## CONFLICT OF INTEREST STATEMENT

The authors declare that they have no competing interests. Author disclosures are available in the Supporting Information.

## CONSENT STATEMENT

We confirm that this study involved no human participants; thus, informed consent was not applicable.

## Supporting information



Supporting Information

Supporting Information
