# Supplementary material for: Chronic cerebral hypoperfusion exacerbates amyloid and tau pathology by impairing glymphatic transport via AQP4‐ and VEGF‐mediated pathways: insights from a vascular to mixed‐type dementia model
Source: Alzheimers Dement. 2026 Mar 17;22(3):e71290. doi: 10.1002/alz.71290 (PMC13093379; doi:10.1002/alz.71290)
Supplement: Supplementary file 1 — Supporting Information [file ALZ-22-e71290-s002.docx]

**Supplementary Materials**

**Table S1.** Time-dependent changes in whole-brain gadobutrol retention

**Table S2A.** Whole-brain gadobutrol wash-in rate

**Table S2B.** Comparison of whole-brain gadobutrol wash-in rates using Dunn’s multiple comparison test

**Table S3A.** Whole-brain gadobutrol washout rate

**Table S3B.** Comparison of whole-brain gadobutrol washout rates using Dunn’s multiple comparison test

**Table S4A.** Whole-brain gadobutrol retention at 300 min

**Table S4B.** Intergroup comparisons of whole-brain gadobutrol retention at 300 min

**Table S5A.** Time-dependent changes in cortical gadobutrol retention

**Table S5B.** Cortical gadobutrol wash-in rate

**Table S5C.** Intergroup comparisons of cortical gadobutrol wash-in rate

**Table S5D.** Cortical gadobutrol washout rate

**Table S5E.** Intergroup comparisons of cortical gadobutrol washout rate

**Table S5F.** Cortical gadobutrol retention at 300 min

**Table S5G.** Intergroup comparisons of cortical gadobutrol retention at 300 min

**Table S6A.** Time-dependent changes in hippocampal gadobutrol retention

**Table S6B.** Hippocampal gadobutrol wash-in rate

**Table S6C.** Intergroup comparisons of hippocampal gadobutrol wash-in rate

**Table S6D.** Hippocampal gadobutrol washout rate

**Table S6E.** Intergroup comparisons of hippocampal gadobutrol washout rate

**Table S6F.** Hippocampal gadobutrol retention at 300 min

**Table S6G.** Intergroup comparisons of hippocampal gadobutrol retention at 300 min

**Figure S1.** BCCAO-induced changes in cerebral blood flow, pulsatility, and resistance.

**Figure S2.** Cortical gadobutrol concentrations and wash-in and washout kinetics.

**Figure S3.** Hippocampal gadobutrol concentrations and wash-in and washout kinetics.

**Figure S4.** BCCAO-induced changes in cell–cell communication revealed by single-cell RNA sequencing.

**Figure S5.** Altered astrocyte–endothelial VEGF signaling following CCH/Aβ exposure.

**Figure S6.** BCCAO reshapes VEGF programs with coordinated changes in Rho-GTPase–cycle genes.

**Table S1.** Time-dependent changes in whole-brain gadobutrol retention

| Minutes | WT-sham  (n = 5) | | WT-BCCAO  (n = 5) | | AD-sham  (n = 5) | | AD-BCCAO  (n = 5) | |
| --- | --- | --- | --- | --- | --- | --- | --- | --- |
|  | Mean | SEM | Mean | SEM | Mean | SEM | Mean | SEM |
| 0 | 1 | 0 | 1 | 0 | 1 | 0 | 1 | 0 |
| 30 | 1.614 | 0.04 | 1.316 | 0.023 | 1.207 | 0.038 | 1.103 | 0.026 |
| 60 | 1.193 | 0.024 | 1.121 | 0.014 | 1.309 | 0.018 | 1.228 | 0.033 |
| 90 | 1.012 | 0.026 | 0.909 | 0.022 | 1.073 | 0.041 | 1.108 | 0.051 |
| 120 | 0.807 | 0.021 | 0.825 | 0.016 | 0.956 | 0.072 | 0.966 | 0.076 |
| 180 | 0.598 | 0.012 | 0.697 | 0.016 | 0.801 | 0.021 | 0.849 | 0.031 |
| 240 | 0.474 | 0.013 | 0.564 | 0.013 | 0.604 | 0.067 | 0.769 | 0.037 |
| 300 | 0.356 | 0.01 | 0.491 | 0.026 | 0.556 | 0.034 | 0.691 | 0.024 |

WT, wild type; BCCAO, bilateral common carotid artery occlusion; AD, Alzheimer’s disease; SEM, standard error of the mean.

**Table S2A.** Whole-brain gadobutrol wash-in rate

|  | WT-sham  (n = 5) | WT-BCCAO  (n = 5) | AD-sham  (n = 5) | AD-BCCAO  (n = 5) |
| --- | --- | --- | --- | --- |
| Mean | 0.06176 | 0.03553 | 0.01794 | 0.003915 |
| Standard deviation | 0.005390 | 0.002340 | 0.002517 | 0.0007313 |
| Standard error of the mean | 0.001205 | 0.0005231 | 0.0005629 | 0.0001635 |

Statistical method: Kruskal-Wallis test.

WT, wild type; BCCAO, bilateral common carotid artery occlusion; AD, Alzheimer’s disease.

**Table S2B.** Comparison of whole-brain gadobutrol wash-in rates using Dunn’s multiple comparison test

| Dunn’s multiple comparison test | Mean rank difference | Significant | Summary | Adjusted  *p* value |
| --- | --- | --- | --- | --- |
| WT-BCCAO versus WT-sham | −20.00 | Yes | * | .039 |
| WT-BCCAO versus AD-BCCAO | 40.00 | Yes | *** | <.001 |
| WT-BCCAO versus AD-sham | 20.00 | Yes | * | .039 |
| WT-sham versus AD-BCCAO | 60.00 | Yes | *** | <.001 |
| WT-sham versus AD-sham | 40.00 | Yes | *** | <.001 |
| AD-BCCAO versus AD-sham | −20.00 | Yes | * | .039 |

WT, wild type; BCCAO, bilateral common carotid artery occlusion; AD, Alzheimer’s disease.

**Table S3A.** Whole-brain gadobutrol washout rate

|  | WT-sham  (n = 5) | WT-BCCAO  (n = 5) | AD-sham  (n = 5) | AD-BCCAO  (n = 5) |
| --- | --- | --- | --- | --- |
| Mean | 0.003757 | 0.002897 | 0.002254 | 0.0009347 |
| Standard deviation | 0.0005249 | 0.0003197 | 0.0003525 | 0.0001056 |
| Standard error of the mean | 0.0001660 | 0.0001011 | 0.00009103 | 0.00002726 |

Statistical method: Kruskal-Wallis test.

WT, wild type; BCCAO, bilateral common carotid artery occlusion; AD, Alzheimer’s disease.

**Table S3B.** Comparison of whole-brain gadobutrol washout rates using Dunn’s multiple comparison test

| Dunn’s multiple comparison test | Mean rank difference | Significant | Summary | Adjusted  *p* value |
| --- | --- | --- | --- | --- |
| WT-BCCAO versus WT-sham | −8.800 | No | ns | >.99 |
| WT-BCCAO versus AD-BCCAO | 27.50 | Yes | *** | <.001 |
| WT-BCCAO versus AD-sham | 11.70 | No | ns | .029 |
| WT-sham versus AD-BCCAO | 36.30 | Yes | *** | <.001 |
| WT-sham versus AD-sham | 20.50 | Yes | ** | .003 |
| AD-BCCAO versus AD-sham | −15.80 | Yes | * | .02 |

WT, wild type; BCCAO, bilateral common carotid artery occlusion; AD, Alzheimer’s disease.

**Table S4A.** Whole-brain gadobutrol retention at 300 min

|  | WT-sham  (n = 5) | WT-BCCAO  (n = 5) | AD-sham  (n = 5) | AD-BCCAO  (n = 5) |
| --- | --- | --- | --- | --- |
| Mean | 18.40% | 31.40% | 34.40% | 58.60% |
| Standard deviation | 2.667 | 4.171 | 2.230 | 1.920 |
| Standard error of the mean | 0.6887 | 1.077 | 0.5757 | 0.4957 |

Statistical method: Kruskal-Wallis test.

WT, wild type; BCCAO, bilateral common carotid artery occlusion; AD, Alzheimer’s disease.

**Table S4B.** Intergroup comparisons of whole-brain gadobutrol retention at 300 min

| Dunn’s multiple comparison test | Mean rank difference | Significant | Summary | Adjusted  *p* value |
| --- | --- | --- | --- | --- |
| WT-BCCAO versus WT-sham | −19.20 | Yes | * | .015 |
| WT-BCCAO versus AD-BCCAO | 25.80 | Yes | *** | <.001 |
| WT-BCCAO versus AD-sham | 6.600 | No | ns | >.999 |
| WT-sham versus AD-BCCAO | 45.00 | Yes | *** | <.001 |
| WT-sham versus AD-sham | 25.80 | Yes | *** | <.001 |
| AD-BCCAO versus AD-sham | −19.20 | Yes | * | .015 |

WT, wild type; BCCAO, bilateral common carotid artery occlusion; AD, Alzheimer’s disease.

**Table S5A.** Time-dependent changes in cortical gadobutrol retention

| Minutes | WT-sham  (n = 5) | | WT-BCCAO  (n = 5) | | AD-sham  (n = 5) | | AD-BCCAO  (n = 5) | |
| --- | --- | --- | --- | --- | --- | --- | --- | --- |
|  | Mean | SEM | Mean | SEM | Mean | SEM | Mean | SEM |
| 0 | 1 | 0 | 1 | 0 | 1 | 0 | 1 | 0 |
| 30 | 8.470593 | 0.526 | 6.033438 | 0.521 | 5.689046 | 0.625 | 4.531519 | 0.891 |
| 60 | 7.169554 | 0.308 | 7.594288 | 0.829 | 7.257301 | 0.571 | 5.351948 | 0.523 |
| 90 | 6.068986 | 0.716 | 7.65705 | 0.527 | 6.383781 | 0.854 | 4.20135 | 0.233 |
| 120 | 5.197479 | 0.992 | 6.091277 | 0.244 | 5.140611 | 0.461 | 4.253848 | 0.101 |
| 180 | 3.877195 | 0.611 | 4.903732 | 0.539 | 4.592521 | 0.172 | 3.827347 | 0.27 |
| 240 | 3.336444 | 0.501 | 3.155338 | 0.735 | 3.007982 | 0.115 | 3.272349 | 0.371 |
| 300 | 2.012136 | 0.303 | 2.383027 | 0.862 | 2.090702 | 0.147 | 2.403595 | 0.415 |

WT, wild type; BCCAO, bilateral common carotid artery occlusion; AD, Alzheimer’s disease; SEM, standard error of the mean.

**Table S5B.** Cortical gadobutrol wash-in rate

|  | WT-sham  (n = 5) | WT-BCCAO  (n = 5) | AD-sham  (n = 5) | AD-BCCAO  (n = 5) |
| --- | --- | --- | --- | --- |
| Mean | 0.1459 | 0.1062 | 0.06589 | 0.01324 |
| Standard deviation | 0.01687 | 0.01319 | 0.005149 | 0.004232 |
| Standard error of the mean | 0.005334 | 0.005898 | 0.001330 | 0.001093 |

Statistical method: Kruskal-Wallis test.

WT, wild type; BCCAO, bilateral common carotid artery occlusion; AD, Alzheimer’s disease.

**Table S5C.** Intergroup comparisons of cortical gadobutrol wash-in rate

| Dunn’s multiple comparison test | Mean rank difference | Significant | Summary | Adjusted  *p* value |
| --- | --- | --- | --- | --- |
| WT-BCCAO versus WT-sham | −7.500 | No | ns | >.999 |
| WT-BCCAO versus AD-BCCAO | 25.00 | Yes | ** | .001 |
| WT-BCCAO versus AD-sham | 10.00 | No | ns | .839 |
| WT-sham versus AD-BCCAO | 32.50 | Yes | *** | <.001 |
| WT-sham versus AD-sham | 17.50 | Yes | ** | .006 |
| AD-BCCAO versus AD-sham | −15.00 | Yes | * | .010 |

WT, wild type; BCCAO, bilateral common carotid artery occlusion; AD, Alzheimer’s disease.

**Table S5D.** Cortical gadobutrol washout rate

|  | WT-sham  (n = 5) | WT-BCCAO  (n = 5) | AD-sham  (n = 5) | AD-BCCAO  (n = 5) |
| --- | --- | --- | --- | --- |
| Mean | 0.03248 | 0.02312 | 0.01537 | 0.006738 |
| Standard deviation | 0.003198 | 0.003185 | 0.001697 | 0.0009867 |
| Standard error of the mean | 0.001011 | 0.001424 | 0.0004381 | 0.0002548 |

Statistical method: Kruskal-Wallis test.

WT, wild type; BCCAO, bilateral common carotid artery occlusion; AD, Alzheimer’s disease.

**Table S5E.** Intergroup comparisons of cortical gadobutrol washout rate

| Dunn’s multiple comparison test | Mean rank difference | Significant | Summary | Adjusted  *p* value |
| --- | --- | --- | --- | --- |
| WT-BCCAO versus WT-sham | −7.500 | No | ns | >.99 |
| WT-BCCAO versus AD-BCCAO | 25.00 | Yes | ** | .001 |
| WT-BCCAO versus AD-sham | 10.00 | No | ns | .84 |
| WT-sham versus AD-BCCAO | 32.50 | Yes | *** | <.001 |
| WT-sham versus AD-sham | 17.50 | Yes | ** | .006 |
| AD-BCCAO versus AD-sham | −15.00 | Yes | * | .01 |

WT, wild type; BCCAO, bilateral common carotid artery occlusion; AD, Alzheimer’s disease.

**Table S5F.** Cortical gadobutrol retention at 300 min

|  | WT-sham  (n = 5) | WT-BCCAO  (n = 5) | AD-sham  (n = 5) | AD-BCCAO  (n = 5) |
| --- | --- | --- | --- | --- |
| Mean | 23.64% | 31.78% | 37.52% | 60.38% |
| Standard deviation | 1.912 | 3.268 | 3.070 | 2.744 |
| Standard error of the mean | 0.6759 | 1.461 | 0.8661 | 0.7921 |

Statistical method: Kruskal-Wallis test.

WT, wild type; BCCAO, bilateral common carotid artery occlusion; AD, Alzheimer’s disease.

**Table S5G.** Intergroup comparisons of cortical gadobutrol retention at 300 min

| Dunn’s multiple comparison test | Mean rank difference | Significant | Summary | Adjusted  *p* value |
| --- | --- | --- | --- | --- |
| WT-BCCAO versus WT-sham | −7.700 | No | ns | >.999 |
| WT-BCCAO versus AD-BCCAO | 19.30 | Yes | ** | .005 |
| WT-BCCAO versus AD-sham | 6.800 | No | ns | >.999 |
| WT-sham versus AD-BCCAO | 27.00 | Yes | *** | <.001 |
| WT-sham versus AD-sham | 14.50 | Yes | * | .020 |
| AD-BCCAO versus AD-sham | −12.50 | Yes | * | .028 |

WT, wild type; BCCAO, bilateral common carotid artery occlusion; AD, Alzheimer’s disease.

**Table S6A.** Time-dependent changes in hippocampal gadobutrol retention

| Minutes | WT-sham  (n = 5) | | WT-BCCAO  (n = 5) | | AD-sham  (n = 5) | | AD-BCCAO  (n = 5) | |
| --- | --- | --- | --- | --- | --- | --- | --- | --- |
|  | Mean | SEM | Mean | SEM | Mean | SEM | Mean | SEM |
| 0 | 1 | 0 | 1 | 0 | 1 | 0 | 1 | 0 |
| 30 | 7.435427 | 0.658285 | 6.181029 | 0.927034 | 5.396977 | 0.833856 | 3.939362 | 0.373714 |
| 60 | 7.297683 | 0.55435 | 5.543277 | 0.501057 | 5.848634 | 0.845103 | 4.047157 | 0.742669 |
| 90 | 5.25316 | 0.391398 | 5.664918 | 0.719971 | 4.715545 | 0.826794 | 3.575664 | 0.945977 |
| 120 | 3.84896 | 0.295401 | 4.225595 | 0.546943 | 3.600192 | 0.554205 | 3.237783 | 0.898204 |
| 180 | 2.579974 | 0.245636 | 3.06284 | 0.334849 | 3.012219 | 0.292571 | 2.822363 | 0.216176 |
| 240 | 2.166725 | 0.181356 | 2.496953 | 0.342371 | 2.664436 | 0.209048 | 2.461209 | 0.191874 |
| 300 | 1.497031 | 0.160902 | 2.125171 | 0.289732 | 2.210651 | 0.200174 | 2.165268 | 0.109875 |

WT, wild type; BCCAO, bilateral common carotid artery occlusion; AD, Alzheimer’s disease; SEM, standard error of the mean.

**Table S6B.** Hippocampal gadobutrol wash-in rate

|  | WT-sham  (n = 5) | WT-BCCAO  (n = 5) | AD-sham  (n = 5) | AD-BCCAO  (n = 5) |
| --- | --- | --- | --- | --- |
| Mean | 0.06731 | 0.04949 | 0.03698 | 0.01415 |
| Standard deviation | 0.003595 | 0.003577 | 0.003938 | 0.003368 |
| Standard error of the mean | 0.0009282 | 0.001601 | 0.001017 | 0.0008697 |

Statistical method: Kruskal-Wallis test.

WT, wild type; BCCAO, bilateral common carotid artery occlusion; AD, Alzheimer’s disease.

**Table S6C.** Intergroup comparisons of hippocampal gadobutrol wash-in rate

| Dunn’s multiple comparison test | Mean rank difference | Significant | Summary | Adjusted  *p* value |
| --- | --- | --- | --- | --- |
| WT-BCCAO versus WT-sham | −10.00 | No | ns | >.999 |
| WT-BCCAO versus AD-BCCAO | 25.00 | Yes | ** | .005 |
| WT-BCCAO versus AD-sham | 10.00 | No | ns | >.999 |
| WT-sham versus AD-BCCAO | 35.00 | Yes | *** | <.001 |
| WT-sham versus AD-sham | 20.00 | Yes | ** | .001 |
| AD-BCCAO versus AD-sham | −15.00 | Yes | * | .029 |

WT, wild type; BCCAO, bilateral common carotid artery occlusion; AD, Alzheimer’s disease.

**Table S6D.** Hippocampal gadobutrol washout rate

|  | WT-sham  (n = 5) | WT-BCCAO  (n = 5) | AD-sham  (n = 5) | AD-BCCAO  (n = 5) |
| --- | --- | --- | --- | --- |
| Mean | 0.006472 | 0.004226 | 0.003664 | 0.0142 |
| Standard deviation | 0.0004783 | 0.0004248 | 0.0002688 | 0.0033 |
| Standard error of the mean | 0.0001235 | 0.0001097 | 0.0000694 | 0.0000762 |

Statistical method: Kruskal-Wallis test.

WT, wild type; BCCAO, bilateral common carotid artery occlusion; AD, Alzheimer’s disease.

**Table S6E.** Intergroup comparisons of hippocampal gadobutrol washout rate

| Dunn’s multiple comparison test | Mean rank difference | Significant | Summary | Adjusted  *p* value |
| --- | --- | --- | --- | --- |
| WT-BCCAO versus WT-sham | −16.80 | Yes | * | .05 |
| WT-BCCAO versus AD-BCCAO | 28.20 | Yes | *** | <.001 |
| WT-BCCAO versus AD-sham | 11.40 | No | ns | .44 |
| WT-sham versus AD-BCCAO | 45.00 | Yes | *** | <.001 |
| WT-sham versus AD-sham | 28.20 | Yes | *** | <.001 |
| AD-BCCAO versus AD-sham | −16.80 | Yes | * | .05 |

WT, wild type; BCCAO, bilateral common carotid artery occlusion; AD, Alzheimer’s disease.

**Table S6F.** Hippocampal gadobutrol retention at 300 min

|  | WT-sham  (n = 5) | WT-BCCAO  (n = 5) | AD-sham  (n = 5) | AD-BCCAO  (n = 5) |
| --- | --- | --- | --- | --- |
| Mean | 19.55% | 38.50% | 44.41% | 60.18% |
| Standard deviation | 2.588 | 4.649 | 2.592 | 1.904 |
| Standard error of the mean | 0.8183 | 1.201 | 0.6693 | 0.4917 |

Statistical method: Kruskal-Wallis test.

WT, wild type; BCCAO, bilateral common carotid artery occlusion; AD, Alzheimer’s disease.

**Table S6G.** Intergroup comparisons of hippocampal gadobutrol retention at 300 min

| Dunn’s multiple comparison test | Mean rank difference | Significant | Summary | Adjusted  *p* value |
| --- | --- | --- | --- | --- |
| WT-BCCAO versus WT-sham | −19.20 | Yes | * | .015 |
| WT-BCCAO versus AD-BCCAO | 25.80 | Yes | *** | <.001 |
| WT-BCCAO versus AD-sham | 6.600 | No | ns | >.999 |
| WT-sham versus AD-BCCAO | 45.00 | Yes | *** | <.001 |
| WT-sham versus AD-sham | 25.80 | Yes | *** | <.001 |
| AD-BCCAO versus AD-sham | −19.20 | Yes | * | .015 |

WT, wild type; BCCAO, bilateral common carotid artery occlusion; AD, Alzheimer’s disease.


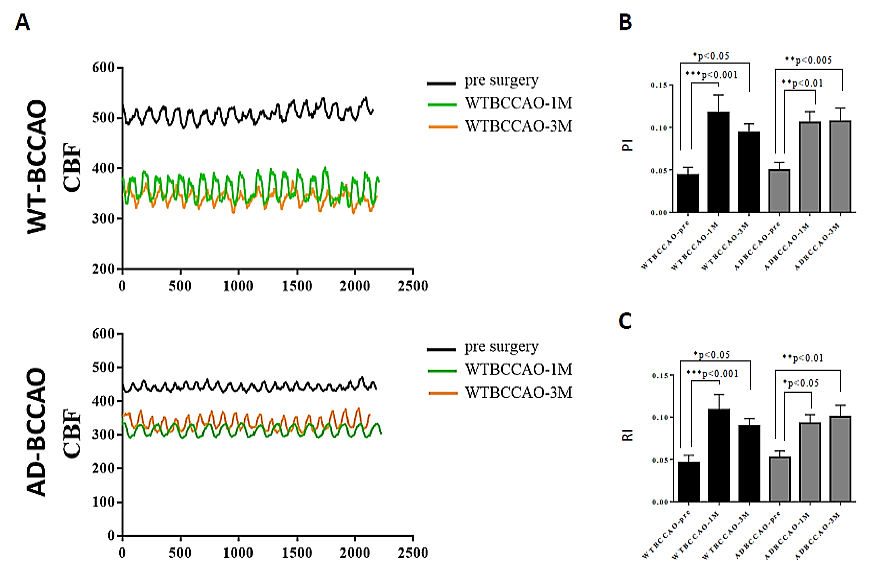


**Figure S1. BCCAO-induced changes in cerebral blood flow, pulsatility, and resistance.**

(A) Cerebral blood flow waveforms in WT and AD mice; the data were obtained through Doppler flowmetry before surgery, and at 1- and 3- month post-surgery. At both 1- and 3-month follow-ups, the (B) cerebral pulsatility index and (C) resistance index values were significantly higher in the WT-BCCAO and AD-BCCAO groups than in the WT-sham and AD-sham groups, indicating sustained vascular dysfunction. **p* < 0.05, ***p* < 0.01, and ****p* < 0.001. BCCAO, bilateral common carotid artery occlusion; WT, wild type; AD, Alzheimer’s disease.

**
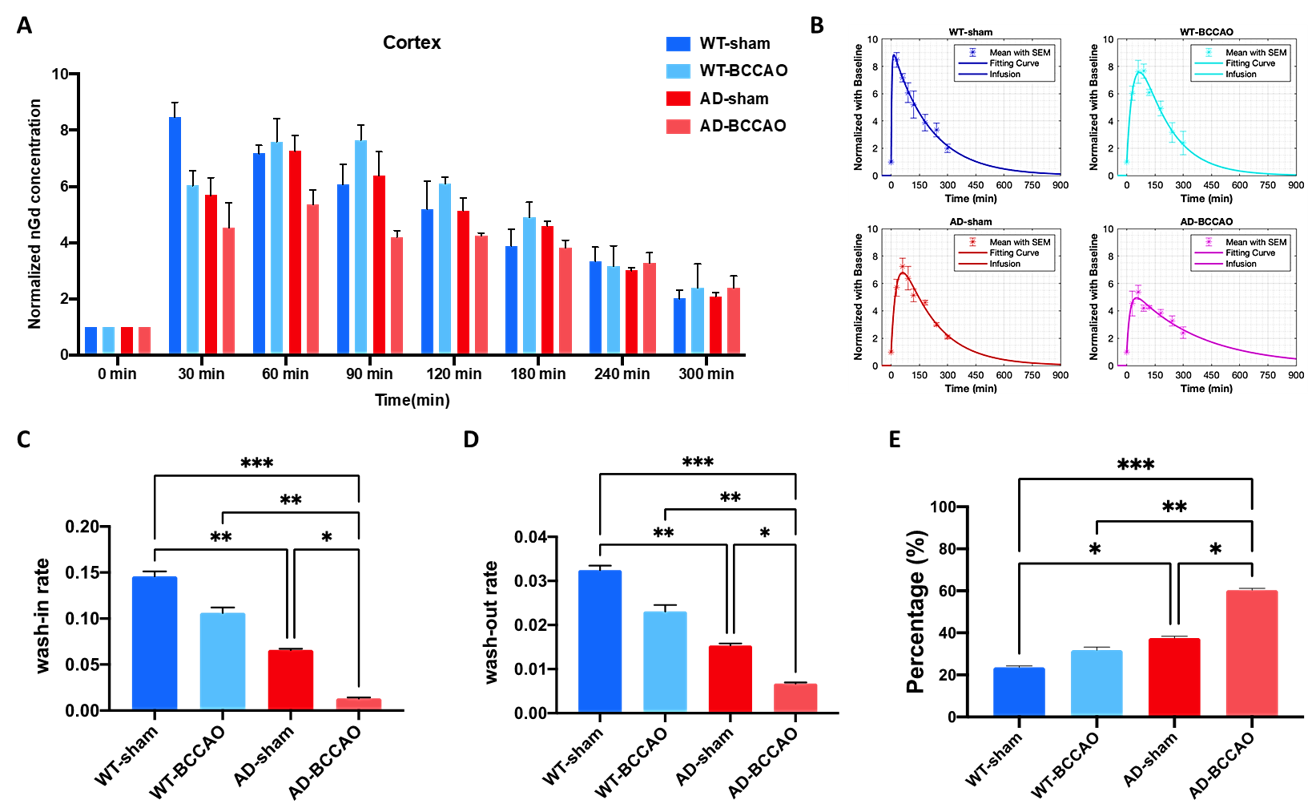
**

**Figure S2. Cortical gadobutrol concentrations and wash-in and washout kinetics.**

Glymphatic function was measured through magnetic resonance imaging with gadolinium injection into the subarachnoid space. (A) Cortical gadolinium concentrations at baseline and 30, 60, 90, 120, 180, 240, and 300 min after injection. Gadolinium reached the highest concentration at 30 min after injection in the WT-sham and WT-BCCAO groups and at 60 min after injection in the AD-sham and AD-BCCAO groups. (B) Curves depicting changes in gadolinium concentration over time: the WT-sham group exhibited the sharpest slopes of increase and decrease, followed by the WT-BCCAO, AD-sham, and AD-BCCAO groups. The rates of (C) wash-in and (D) washout and (E) the proportion of gadolinium remaining at 300 min; the findings indicated that both BCCAO and AD impaired glymphatic function. **p* < 0.05, ***p* < 0.01, and ****p* < 0.001. WT, wild type; BCCAO, bilateral common carotid artery occlusion; AD, Alzheimer’s disease.


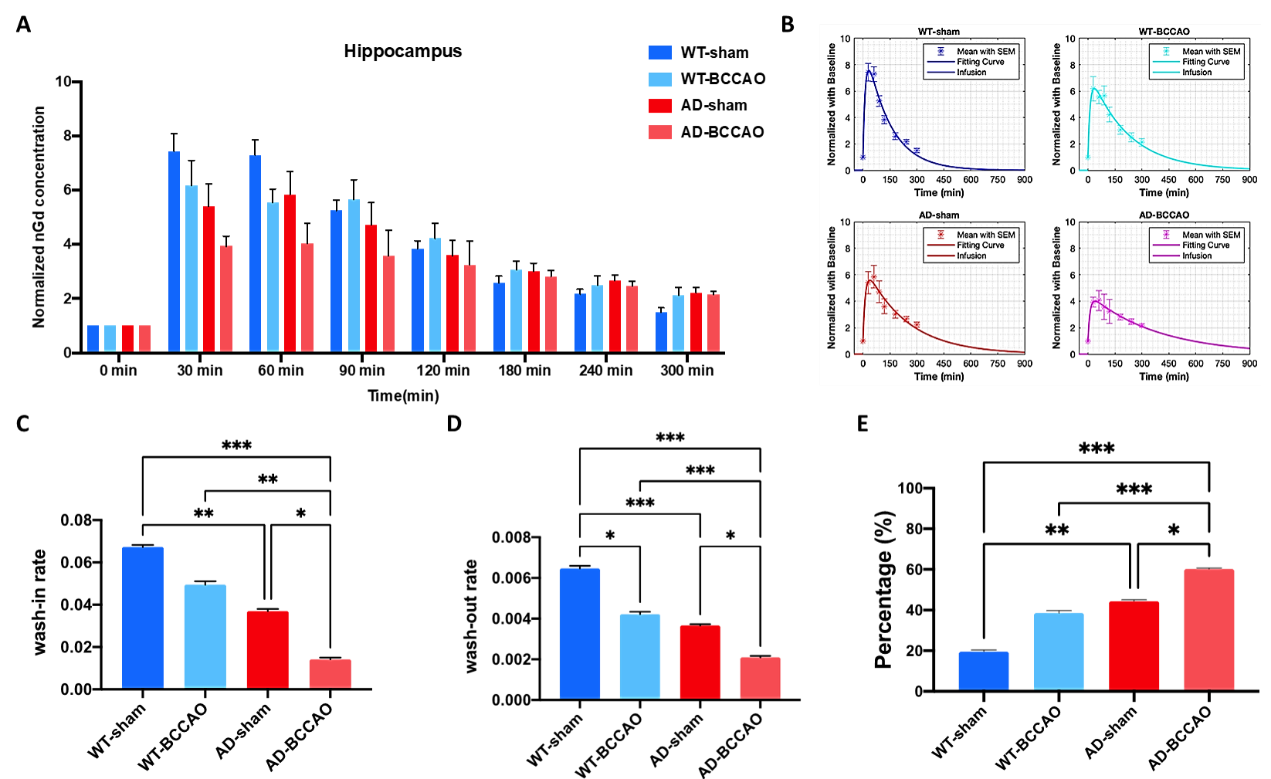


**Figure S3. Hippocampal gadobutrol concentrations and wash-in and washout kinetics.**

Glymphatic function was measured through magnetic resonance imaging with gadolinium injection into subarachnoid space. (A) Hippocampal gadolinium concentrations at baseline and 30, 60, 90, 120, 180, 240, and 300 min after injection. Gadolinium reached the highest concentration at 30 min after injection in the WT-sham and WT-BCCAO groups and at 60 min after injection in the AD-sham and AD-BCCAO groups. (B) Curves depicting changes in gadolinium concentration over time: the WT-sham group exhibited the sharpest slopes of increase and decrease, followed by the WT-BCCAO, AD-sham, and AD-BCCAO groups. The rates of (C) wash-in and (D) washout and (E) the proportion of gadolinium remaining at 300 min; the findings indicated that both BCCAO and AD impaired glymphatic function. **p* < 0.05, ***p* < 0.01, and ****p* < 0.001. WT, wild type; BCCAO, bilateral common carotid artery occlusion; AD, Alzheimer’s disease.

**
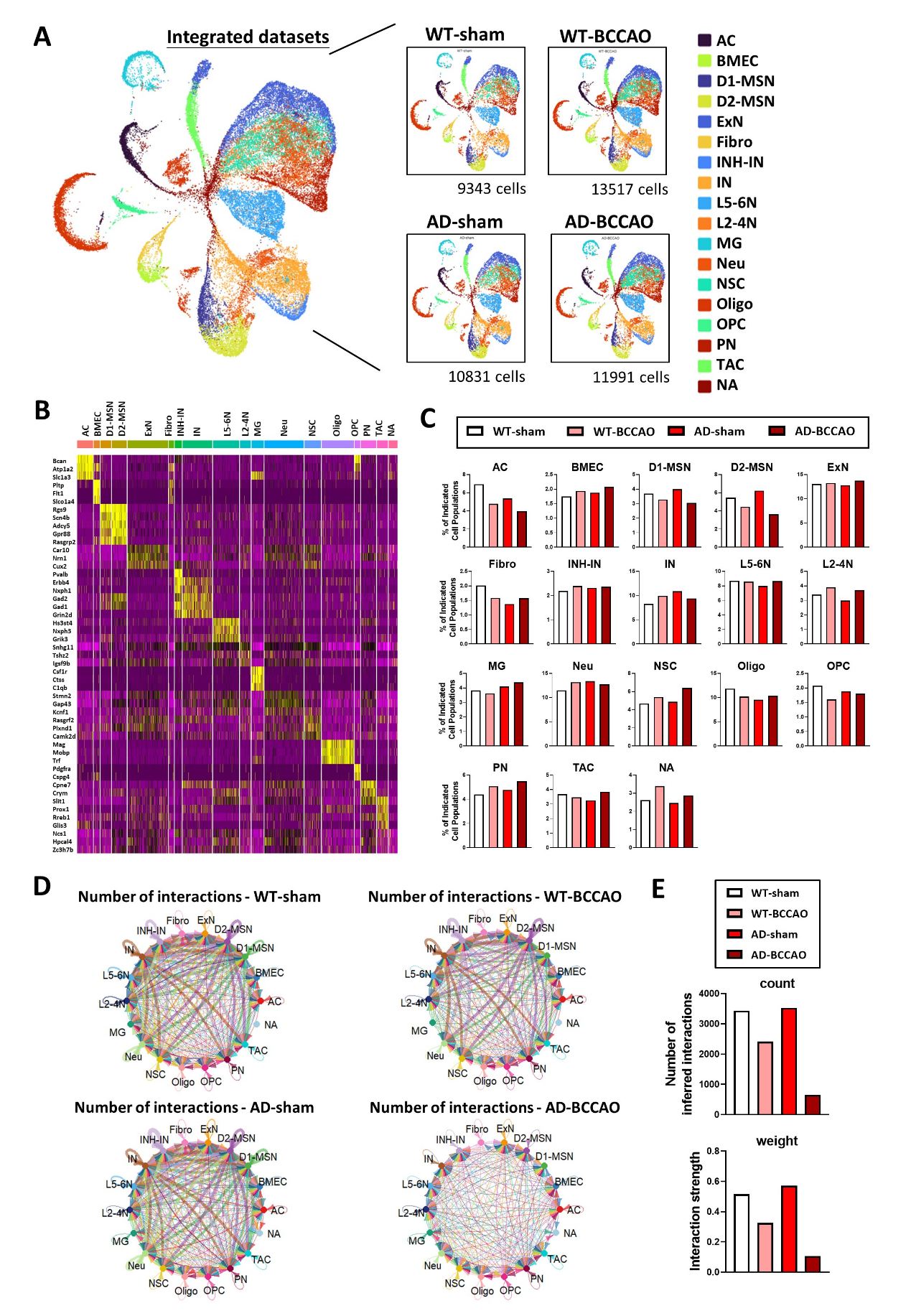
**

**Figure S4. BCCAO-induced changes in cell–cell communication revealed by single-cell RNA sequencing.**

(A) UMAP visualization of the integrated dataset (left), colored by annotated cell classes; condition-specific UMAPs (right) display cell-type distributions for WT-sham (9,343 cells), WT-BCCAO (13,517 cells), AD-sham (10,831 cells), and AD-BCCAO (11,991 cells) groups. (B) Heatmap depicting canonical marker genes used for cell-type annotation across major cell classes. (C) Bar plots showing the fraction of cells assigned to each class in the four conditions, highlighting condition-specific shifts in cellular composition. (D) Circle plots summarizing the number of inferred ligand–receptor interactions between cell classes for each condition; edge density corresponds to interaction counts. (E) Global summaries of intercellular communication, showing the total number of inferred interactions (count) and the aggregated interaction strength (weight) per condition. AC, astrocytes; BMEC, brain microvascular endothelial cells; D1-MSN/D2-MSN, D1/D2 dopamine receptor–expressing medium spiny neurons; ExN, excitatory neurons; IN, interneurons; INH-IN, inhibitory interneurons; L2–4N and L5–6N, layer-specific cortical neurons; MG, microglia; Neu, neurons; NSC, neural stem cells; Oligo, oligodendrocytes; OPC, oligodendrocyte precursor cells; PN, pyramidal neurons; TAC, transit-amplifying cells; NA, not assigned. UMAP, uniform manifold approximation and projection; WT, wild type; BCCAO, bilateral common carotid artery occlusion; AD, Alzheimer’s disease.


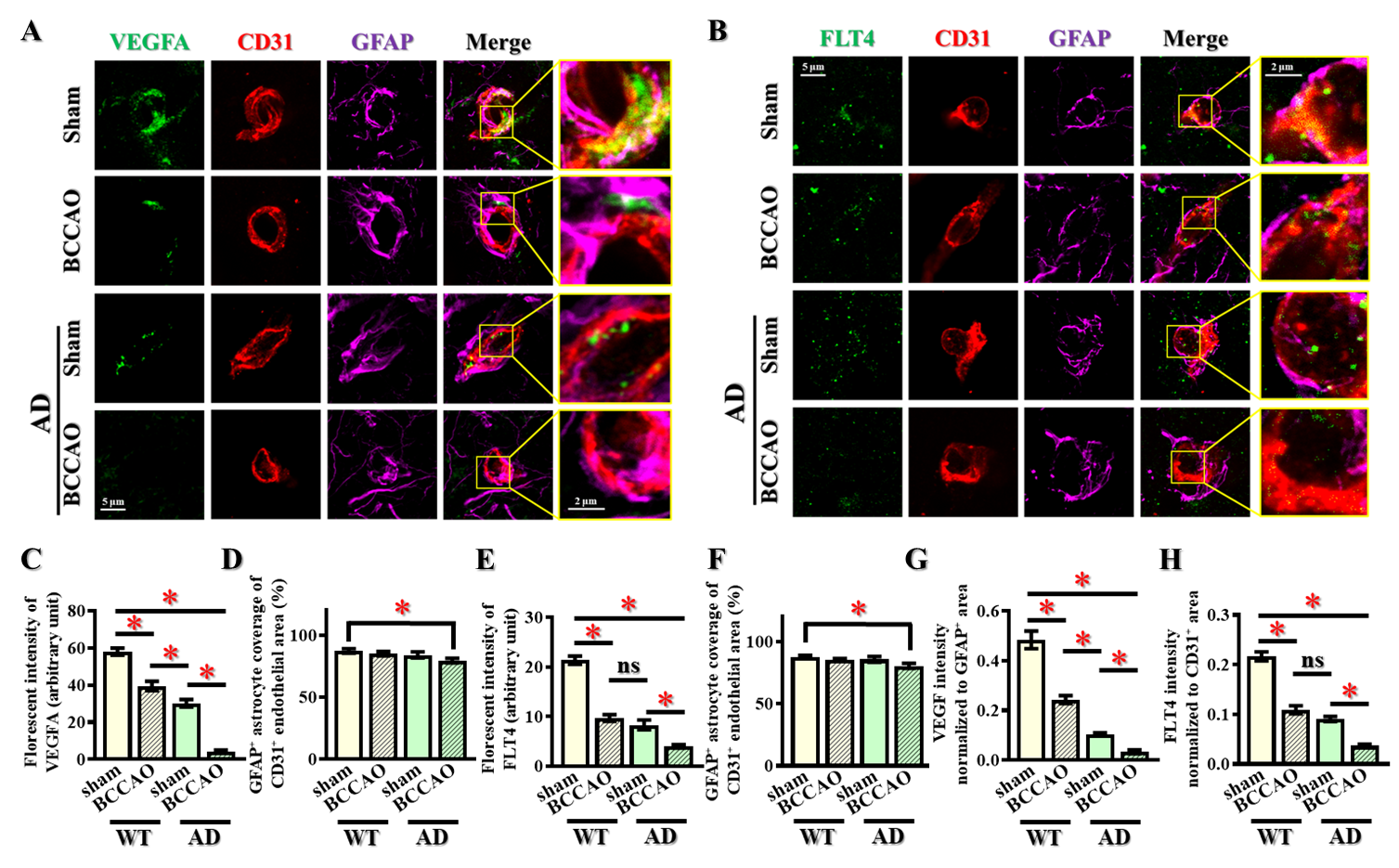


**Figure S5. Altered astrocyte–endothelial VEGF signaling following CCH/Aβ exposure.**

(A) Representative immunofluorescence images showing VEGFA (green), CD31 (red), and GFAP (magenta) signals in the indicated experimental groups. (B) Representative immunofluorescence images showing FLT4 (green), CD31 (red), and GFAP (magenta). (C) Quantification of VEGFA fluorescence intensity from A. (D) Quantification of astrocyte–vessel association, expressed as the percentage of CD31⁺ vessel length covered by GFAP⁺ astrocytic processes. (E) Quantification of FLT4 fluorescence intensity from B. (F) Quantification of astrocyte–vessel association in the same regions of interest. (G) VEGFA fluorescence intensity normalized to GFAP⁺ astrocytic area. (H) FLT4 fluorescence intensity normalized to individual CD31⁺ endothelial cells. VEGF, vascular endothelial growth factor; CCH, chronic cerebral hypoperfusion; Aβ, amyloid beta; WT, wild type; AD, Alzheimer’s disease; BCCAO, bilateral common carotid artery occlusion; GFAP, glial fibrillary acidic protein; CD31, platelet endothelial cell adhesion molecule-1, PECAM-1; FLT4, vascular endothelial growth factor receptor-3, VEGFR-3.


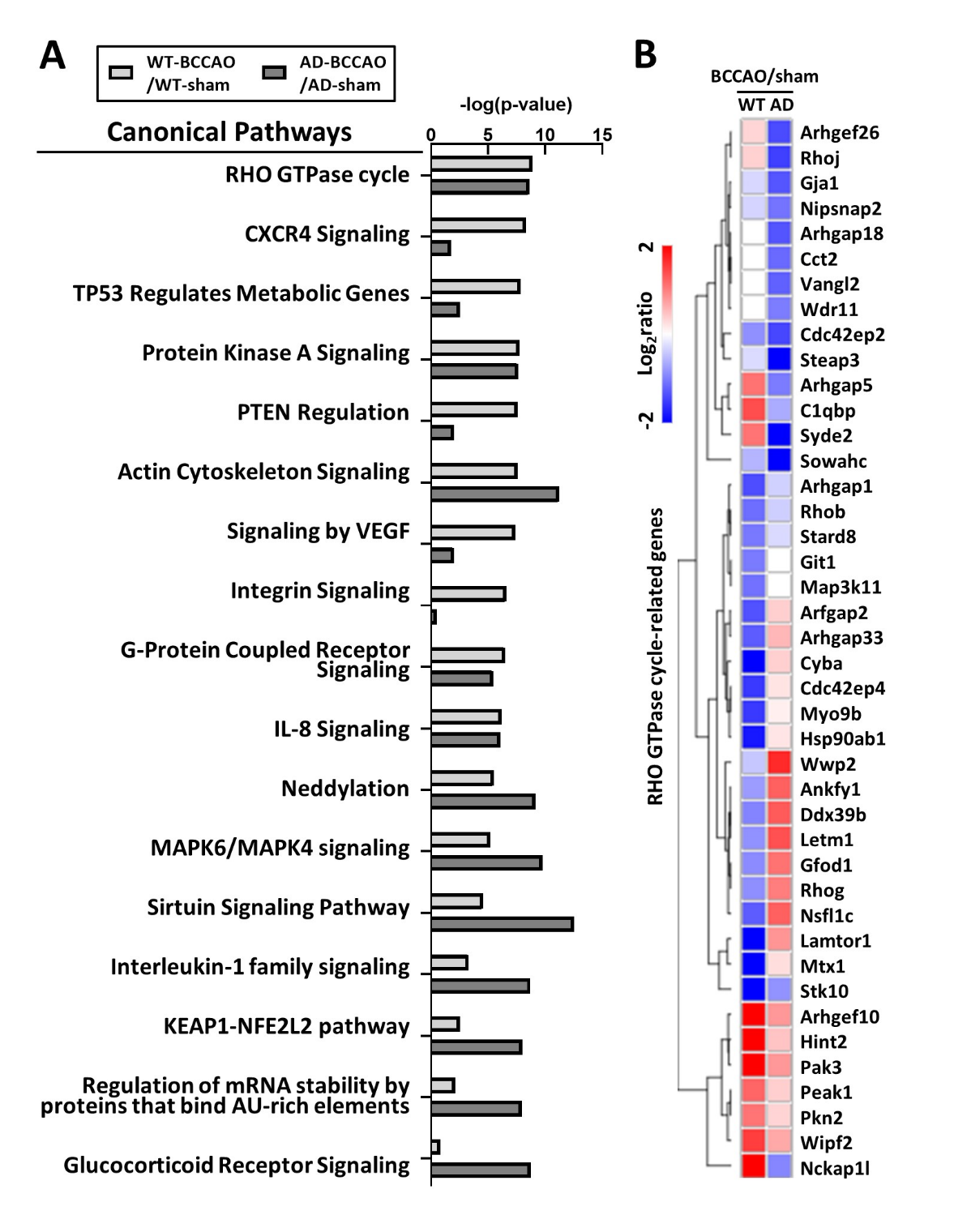


**Figure S6. BCCAO reshapes VEGF signaling programs with coordinated changes in Rho-GTPase–cycle genes.**

(A) Canonical pathway enrichment analysis of differentially expressed genes (BCCAO vs. sham) in BMEC from WT and AD brains, performed using Ingenuity Pathway Analysis (IPA); bars represent −log(p-value). (B) Heatmap with hierarchical clustering of curated Rho-GTPase cycle–related genes showing log2 fold changes (BCCAO/sham) in BMEC from WT and AD brains; red indicates upregulation, blue indicates downregulation. BCCAO, bilateral common carotid artery occlusion; VEGF, vascular endothelial growth factor; BMEC, brain microvascular endothelial cells; WT, wild type; AD, Alzheimer’s disease.
